# Observation inflation as source confusion: Symmetrical conflation of memories based on action performance and observation

**DOI:** 10.1177/17470218241306743

**Published:** 2024-12-27

**Authors:** Bence Neszmélyi, Roland Pfister

**Affiliations:** 1 Department of Psychology III, University of Würzburg, Würzburg, Germany; 2 Department of Psychology, Trier University, Trier, Germany; 3 Institute for Cognitive and Affective Neuroscience (ICAN), Trier University, Trier, Germany

**Keywords:** Observation inflation, action memory, motor simulation, source monitoring

## Abstract

People often cannot remember the source of their memories despite recalling other elements of a remembered event correctly. Observation inflation is one such error of source monitoring. It refers to remembering the actions of another agent as self-performed. While the existence of this memory error is well documented, it is not clear how it relates to other errors of source attribution: It is not evident whether the phenomenon reflects (1) a specific tendency to appropriate the actions of other agents, (2) a general confusion of sources with overlapping features, or (3) whether it is a confound induced by the complex structure of the conventionally used experimental paradigm. We conducted two online experiments to assess these potential contributions to observation inflation. Crucially, administering a full source monitoring test revealed a symmetrical pattern: Recognising other’s actions as one’s own occurred at the same rate as misattributing one’s own actions to another agent. The findings resonate with source-monitoring frameworks by suggesting that source attribution errors arise due to the similarity of the sources, whereas the evidence speaks against a special status for appropriating observed actions.

## Introduction

### Source monitoring errors

Human memory is notoriously fallible. We are constantly reminded of this when trying to recall previous information or events (McDermott & Roediger, 1998; Schacter et al., 2011; Wells & Loftus, 2003). Even more shocking is to realise that eventually recalled memories of our own actions are also unreliable: They are susceptible to source memory errors (Leynes & Kakadia, 2013; Manzi & Nigro, 2008) in that we tend to confuse memories of self-performed actions with memories originating from another source. This can have critical consequences if memories pertain to events like locking the door or taking medication: We sometimes remember performing such tasks even though we had only imagined performing them (*imagination inflation*; Garry et al., 1996; Goff & Roediger, 1998).

Observing (rather than imagining) an action can also induce false memories of self-performance (*observation inflation*; Foley et al., 1993; Lindner et al., 2010; Sommerville & Hammond, 2007). Conflicts about author contributions in a study may serve as evidence that observation inflation also occurs outside the laboratory. However, while it is clear that observing an action can result in memories of actually performing the same action, the prerequisite conditions of this self-attribution error and its scientific implications are debated. A reason for this might relate to the complex structure of the experimental paradigm that is commonly used to examine observation inflation, rendering it difficult to identify what the phenomenon actually represents. It has been often interpreted as a specific self-attribution error induced by the misinterpretation of motor cues during action observation (Lindner et al., 2010, 2016; Wang et al., 2022). Some findings, however, indicate that it reflects a more general tendency to confuse events with similar sources (Lange et al., 2017), and it is even possible to explain the phenomenon in terms of general memory processes and response tendencies without a reference to source memory. To isolate the influence of these factors, we created a maximally neutral experimental setup for the current study, combined with a comprehensive memory test to control for biases that potentially affected previous results.

### What is observation inflation?

Studies in the last decade often used an experimental protocol that we label the *three-phase paradigm* here (Lindner et al., 2010). In this paradigm, experiments start with an initial *presentation phase*. During this phase, participants receive a set of action statements. Each statement is randomly assigned to one of two trial types: One requires participants to act on the statements (e.g., shaking a bottle), in the other, statements are only read by the participants without executing the action (e.g., reading the sentence “Shake bottle.”). The following *observation phase* is held directly after the first one. During this part, the same set of statements is used as in the first phase, complemented by a few statements that had not been presented previously. For half of the set, participants observe another agent perform the action described in the statements while the other half of the statements is not presented in any form in this phase. The third phase is a surprise *memory test* that follows after a retention interval of several days. Participants have to recognise which actions they had performed in the first phase. The test exclusively focuses on the presentation of the action statements in the initial presentation phase. This is usually done by showing statements presented in the first two phases, intermixed with new statements that were not presented in any of the previous stages. Participants are then asked to decide whether they had performed the action in question in the initial phase. In the original version of the experiment (Lindner et al., 2010) participants were presented with a binary option for this test (“Yes, I did perform the action.” vs “No, I did not perform the action”). In some later versions (e.g., Lindner et al., 2016; Wang et al., 2022), the range of response options was extended (“performed,” “read,” “not presented”). The findings consistently show that action observation increases the probability of actions being reported as self-performed, even if participants did not actually perform them.

Interpretations of observation inflation often draw on the source monitoring framework (Johnson et al., 1993) and state that action observation is misattributed to performance during retrieval because memories of observed actions are characterised by features that are generally considered cues of self-performance. The difference between various theories lies in what they identify as misinterpreted self-performance cues. The predominant explanation is the *motor simulation* account (Lindner et al., 2010, 2016; Wang et al., 2022). Several studies indicate that observing another agent executing a movement triggers the same internal motor programmes as performing the action (see Grèzes & Decety, 2001; Rizzolatti & Craighero, 2004 for reviews). This vicarious activation of the motor system results in motor codes of self-performance being associated with the representation of the observed action. When recalling memories of the observed actions, the automatic reactivation (Leynes & Bink, 2002; Masumoto et al., 2006) of self-performance cues integrated with the representations can result in the misattribution of observed actions to self-performance (Lindner et al., 2016).

Alternative accounts explain the misattribution of observed actions by different cues of self-performance, namely anticipatory processes involved in action planning or perceptual features that characterise action execution. The *shared cognitive operation* account is based on studies that investigated the appropriation of observed actions in cooperative tasks (e.g., two agents building toys or completing puzzles together; Foley et al., 2002; Foley & Ratner, 1998; Sommerville & Hammond, 2007). These indicate that similar anticipatory processes play a role in performing actions as well as in observing actions, which can result in the conflation of sources. This explanation is similar to the motor simulation account since both rely on the assumption that there is a substantial overlap in motor mechanisms responsible for action observation and action performance. However, while the shared cognitive operation theory presumes that anticipatory processes that are typical for self-performed actions only play a role in action observation under specific circumstances (e.g., common goal, turn-taking: Foley et al., 2002; Ratner et al., 2002), the motor simulation account presupposes an automatic activation of motor codes by action observation. In contrast to these two theories that are based on overlapping motor processes, a third account suggests that perceptual features of observed actions are interpreted as cues of self-performance, due to the *perceptual similarity* of the two event types. Perceptual and sensory details like colour of the manipulated object or the sound of the action can be encoded both when performing and when observing actions. Vivid, perceptually rich representations are usually attributed to self-performance (Thomas et al., 2003); thus, the presence of such characteristics might lead to the misattribution of action observation to performance (Lindner et al., 2016).

While self-attribution errors induced by action imagination have often been explained by the perceptual similarity of imagined and performed actions (Johnson et al., 1988; Thomas et al., 2003), studies using the three-phase paradigm to assess action observation did not support such explanations (Lindner et al., 2010, 2016). Instead, experiments in which participants performed a motor task during action observation showed reduced observation inflation if the additional motor task interfered with the motor component of the observed action. This has been interpreted as evidence in support of the motor simulation account (Lindner et al., 2016; Wang et al., 2022).

It is questionable, however, if the three-phase paradigm is indeed assessing the targeted phenomenon. In these studies, performance and observation occur in two separate phases of the experiment. This particular design choice might bias the results in several ways. Here we highlight two possible concerns that are associated with this experimental design.

1. *The response options of the memory test are not consistent with participants’ experience during the task.*

The three-phase paradigm assumes a blurring of the first two phases (initial presentation and observation): Action observation occurs in the second phase, but according to the theory, the observed actions are remembered as if they had been performed in the first phase. The source monitoring tests which are used in three-phase studies are, however, incompatible with this line of reasoning, because they suggest a clear separation of the two action presentation stages. These tests specifically target memories of the first phase, and they only present sources among the response options that were actually featured in that phase (i.e., performed/not performed or performed/read/not presented). That is, participants cannot report that they remember observing an action in the first phase, which is in contradiction with the presumed blurring of the two phases. This setup renders straightforward interpretation of the participants’ responses difficult. When participants categorise an action statement as one that they had performed in the first phase, it is questionable whether they make this selection because they actually remember the event this way, or because none of the presented options reflects their experience, and they are forced to select the closest one from the available options. In the latter case, observation inflation would not tap into source memory errors but actually measure response tendencies in a situation without adequate options.

2. *Results obtained with the three-phase paradigm do no show whether observation inflation is an egocentric effect.*

A second issue with the three-phase paradigm is that while it assumes that observation inflation is an egocentric effect (Lange et al., 2017; Pfister et al., 2017), this presumption is actually not addressed by the method. Thus, it is possible that the effect is based on the bidirectional conflation of action sources (i.e., self-performance and observation) without memories gravitating particularly strongly towards self-performance. To show an egocentric nature of the observation inflation effect, an experimental design is required that also assesses the possibility of a reverse effect (i.e., *self-performance inflation effect*: whether performing an action increases the probability of remembering that action as having been performed by another agent). Although this cannot be done with the conventional three-phase design, some related experimental methods have addressed the question. The results are ambiguous: In Experiment 1 of Lange et al. (2017), a group recalling self-performed actions made substantially more self-attribution errors (recognising the partner’s actions as self-performed) compared to the reverse errors of a group recalling observed actions (recognising own actions as actions performed by the partner). In Experiment 2, however, this finding was not replicated,^1^ and other studies either reported no difference between these two error types or they even observed a larger frequency of self-performed actions being attributed to the other agent (Conway & Dewhurst, 1995; Manzi & Nigro, 2008). Studies that investigated the appropriation of others’ actions in cooperative settings reported that this source error type is more frequent than the opposite error of categorising self-performed actions as actions performed by the co-actor (Foley et al., 1993, 2002; Ratner et al., 2002; Sommerville & Hammond, 2007). However, this effect was only observed in preschool and early school-age children, in older populations there was no evidence for source monitoring errors gravitating towards self-performance.

### The present study

The goal of the current study was to investigate observation inflation with an experimental paradigm that straightforwardly addressed the above issues. This was done in two steps: In Experiment 1, we examined whether we could replicate previous findings of observation inflation with an experimental paradigm that does not separate performance and observation stages. At this stage, we retained the memory test with the binary decision task that was used in the first observation inflation studies (“performed” vs “not performed”; e.g., Lindner et al., 2010). In Experiment 2, the simplified experimental structure was combined with a full source monitoring test that allowed us to compare self-attribution errors induced by action observation to other types of source-monitoring errors and, thus, to assess if observation inflation indeed reflects a specific tendency to appropriate others’ actions or if it can be explained by more general memory processes.

## Experiment 1

In Experiment 1, we simplified the structure of the original three-phase paradigm, eliminating possible confounds stemming from item memory^2^ and temporal misattribution that could be related to the multiple presentation stages. We retained the presentation modes of the original paradigm (perform, observe, and read) but they were all included in a single session. This way, we could compare the conflation of self-performance with an action-related source (observing others’ actions) and with an external source not related to action (reading).

Because the study was conducted during the COVID-19 pandemic, we developed an online procedure. Besides the theoretically motivated adjustments of the experimental design, these circumstances resulted in further deviations from previous methods: (1) In the original version, participants performed a wide range of actions, and each action was specific for the object that was manipulated (e.g., shaking bottle, rolling dice). Operations in a virtual environment are much more constrained. Thus, in the current experiment, participants performed similar actions in each trial; that is, they collected an object from different locations on the screen and navigated it into a box. The only aspect that varied was the object that was being moved. (2) In online studies, it can be difficult to convince participants that they are interacting with a real human partner. Thus, we decided to inform them that their co-actor is an artificial agent. (3) In the original experiment, the memory test was administered 2 weeks after the presentation phases. Pilot studies indicated that with the same delay, participants did not perform above the chance level in the online experiment. Thus, in the current version, the test was performed the day after the first session (see Lange et al., 2017, or Sommerville & Hammond, 2007 for similar, or even shorter retention intervals). We also adjusted the number of presented actions, so that participants could perform reasonably well on the memory test. (4) To ensure that participants paid attention to the presented items, we added attention checks in the presentation phase.

Studies that investigated observation inflation in cooperative situations suggested that prospective processes, like planning and anticipation, play a central role in the appropriation of observed actions (Foley et al., 2002; Ratner et al., 2000). Furthermore, such processes also contribute to a better overall memory of observed events (Zimmer & Engelkamp, 1984). To increase the role of such processes in our experiments, we used a version with commanded actions as a starting point for our method (Pfister et al., 2017). In this version, instead of passive observation, participants instruct the other agent to perform the actions. This approach has also been used successfully for assessing self-attribution errors in a cooperative experimental design (Ratner et al., 2002).

In Experiment 1, our goal was to assess whether we could replicate the observation inflation effect with the new design. Thus, instead of the complete source monitoring test that we applied in Experiment 2, we used the same binary decision task that was applied in the original version of the three-phase paradigm (Lindner et al., 2010) and also in a similar version with commanded actions (Pfister et al., 2017): Participants were asked whether they had performed the action themselves in the action presentation phase, but they did not have to identify the source of actions that they recognised as “not performed.” An additional second test round was also included to specify the source of actions remembered as “not performed.” However, since the second-round responses are conditional on the first test round, we report the analysis of the second test round in the Supplementary Material.

### Method

#### Participants

Our experimental design differed in several aspects from previous studies (online data collection, shorter retention interval, within-subject design) so we decided not to use prior results to determine the required sample size. To remain conservative, we therefore assumed an effect size of *d_z_* = 0.4 (as compared to *d_z_* = 1.89 for Experiment 1 in Pfister et al., 2017). Power analysis indicated that 51 participants would be required to reveal such an effect with 1-β = .8 power at a significance threshold α = .05 using a two-tailed *t*-test for paired samples. (In the present experimental design, the observation inflation effect can be revealed by comparing percentages of erroneous “performed” responses for observed actions and for action statements that were only read.) Considering this target sample size, expected drop-out, and rejection rates, based on prior experience with online data collection, we recruited 75 participants on the Prolific website. This initial sample size and further methodological decisions were preregistered at https://aspredicted.org/KJ4_QT6.

All participants were native English speakers, and they were all right-handed. Six participants failed the attention check (recognising mismatch trials) and were not invited to the second session. Furthermore, nine participants chose not to participate in the second part of the study, thus, the second session was completed by 60 participants. Eleven of these participants did not meet the inclusion criteria (they could not reliably distinguish new items and items that they had encountered in the first session) and were excluded from the analyses. The final sample consisted of 49 participants (mean age: 26.8 years, range: 18-35 years, 19 men, 26 women, 4 chose the “prefer not to say” option). This sample size is sufficient to reveal an effect of the magnitude *d_z_* = 0.41 with the parameters described above and provides a power of 1- β > .99 for the effect size observed in Experiment 1 of Pfister et al. (2017). Participants received payment for completing each of the sessions (£3.50 for the first and £1.00 for the second session). In both sessions, participants were able to obtain a bonus if they performed well (a maximum of £1.00 for the first and £2.00 for the second session). At the start of the experiment, they were informed about the procedure, and they approved a consent form before each session. The experimental procedure was in accordance with the Declaration of Helsinki, and it was approved by the ethics committee of the University of Würzburg.

#### Experimental design

The study consisted of two experimental sessions: In the first session, participants encountered 24 action statements in three different conditions. The second session followed a day later and featured a recognition task concerning the action statements of the previous session. The three conditions for the presentation of action statements in the first experimental session were arranged in a within-subject design. In the *perform condition*, participants performed the presented action, in the *command condition*, they commanded an artificial agent to perform the action, and they observed as it was executed. In the *read condition*, participants only had to read the action statement. In the second session, participants completed a source memory (recognition) test and had to decide whether they had performed the action themselves in the first phase. After that, in the second round, we presented those items that a participant did not remember as performed, asking which presentation condition those actions had been assigned to.

#### Stimuli

The item pool comprised 32 action statements. Each action statement described the act of collecting an object (represented by the image of the object), by moving it into a box icon located at the bottom of the screen (e.g., *Put the ball in the box!*). The action statements only varied with regard to the object that had to be moved. The drag-and-drop actions that had to be executed were identical for each statement. For each statement, images of easily recognisable everyday objects were chosen from the image set used in Pfeuffer et al. (2017; adapted from Brady et al., 2008 and Moutsopoulou et al., 2015). Besides the 32 target objects (i.e., objects referenced in the statements), an additional 66 objects were chosen as distractor items, which were presented together with the target objects during the experimental trials. Both target and distractor objects were presented in colour, with rectangular white background. The size of the target and distractor images was adjusted to 0.13 × 0.13 height units.^3^ The size of the hand icon that was used for the drag and drop actions was 0.05 × 0.05 height units, and the size of the box icon 0.20 × 0.10 height units. The action statements were presented in white colour with a font size of 0.05 height units. The experiment ran in a window with a dark grey background colour.

#### Task

**Figure 1.**
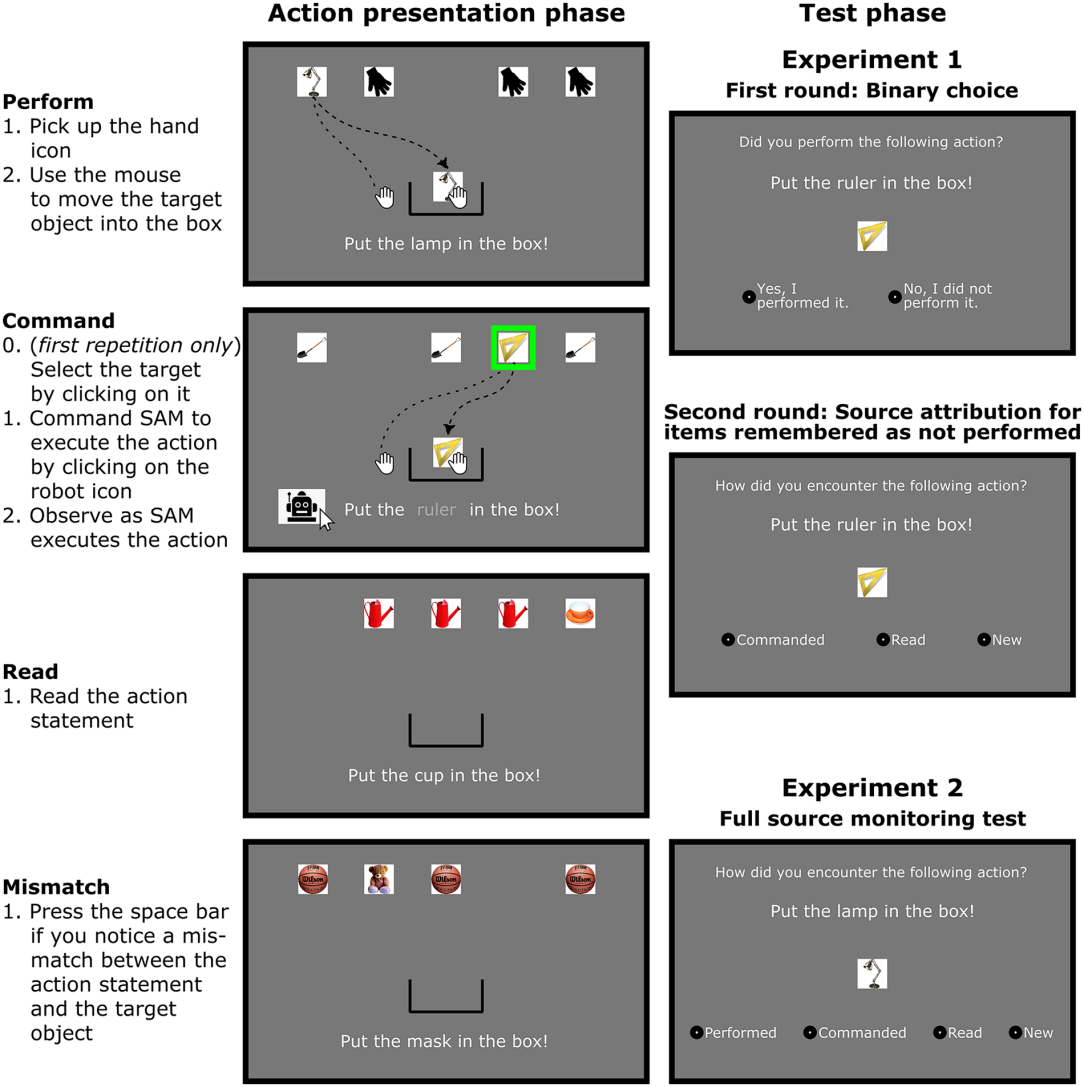
Tasks in the action presentation phase (left) and the test phase (right). The action presentation phase was identical in the two experiments, with three main conditions (perform, command, read) and catch trials (mismatch). In Experiment 1, the test phase was divided into two rounds. In round 1, participants responded whether they performed the action themselves. Items that they categorised as “not performed” were presented again in round two, where participants were asked to specify whether/how they had encountered the item in the action presentation phase. In Experiment 2, the test phase only consisted of a single round, in which a full source monitoring test was administered. A video recording of the action presentation phase and the test phases is available as Supplementary Material.

The task consisted of an action presentation phase and a test phase. Figure 1 gives an overview of the tasks in both of these phases.

##### Action presentation phase

For each participant, we randomly selected 24 action statements from the item pool to be presented in the first session whereas the remaining 8 action statements were not featured in the first session. The 24 selected action statements were randomly assigned to one of the three action presentation conditions. The action statements were presented twice during the experiment, resulting in two runs of 24 experimental trials (48 trials in total). The order of the action statements was randomised in both runs. Each trial started with a screen displaying the current condition—*perform, command* or *read*. To make sure that participants were prepared to perform the required action, icons representing the three options were displayed on the screen and participants had to select the one corresponding to the instruction. After trial initiation, an empty screen was displayed for 500 ms. Following that, the action statement appeared on screen, accompanied by a box icon, the target object, and three distractor objects. The three distractor objects were identical image stimuli. In each trial, the target object was sampled randomly (without replacement) from the pool of 24 remaining target objects, while the distractor object was from the separate pool of 66 distractor objects (also sampled randomly without replacement). The box icon always appeared in the same starting position in the lower half of the screen, and the action statement was displayed below the box. The four objects appeared at the top of the screen, aligned in a horizontal line. Five equally distanced positions were available for the objects and four of them were randomly assigned to the four objects to be displayed on screen (one target and three distractors).

In *perform trials*, a hand icon was displayed next to the box. Participants first had to pick up the hand icon by moving the cursor over it and left-clicking with the mouse. The hand icon then followed the movement of the cursor. Participants then could move to the target object and pick it up by pressing down the left mouse button. They had to hold down the button to drag the object into a box area in the bottom centre of the screen. If the button was released, the object stopped moving but could be picked up again. Upon moving the target object over the box icon, the distractor objects disappeared from the screen, signalling the successful completion of the action. Then, for 1500 ms, only the target object, the hand, and the box icon remained on the screen, but during this time, none of the objects could be moved. After that, the screen was empty for 500 ms. The action had to be repeated four times in a trial. During the four repetitions, the starting location of the hand icon and the position of the box icon were always identical. However, the location of the target and distractor objects was randomly selected for each repetition from the five possible positions. The identity of the target and distractor stimuli was constant across a trial. However, in the two runs, a different distractor was paired with the same target object. (Each distractor item appeared in only one trial during the experiment.)

In *command trials*, participants saw the box, the hand icon, the target, and the distractor objects on the screen. The location of the stimuli was determined in the same way as during the perform trials. The action statement was also displayed below the box, but it was formulated as a command to the artificial agent (introduced as “SAM” during the instructions), and the name of the target object was omitted (e.g., *SAM, put the . . . in the box!*). A robot icon representing SAM was also displayed next to the box. Participants received the instruction that the artificial agent was controlling the cursor, and it would only execute the action if it was commanded by the participant to do so. Participants could instruct SAM to execute the action by clicking first on the target object and then on the robot icon. When clicking on the target object, the name of the target appeared in the action statement, and the click on the robot icon finalised the command, and submitted it to SAM. (If participants clicked on one of the distractor objects, an error message appeared on the screen, instructing participants to select the right target.) After finalising the command, the action statement changed to the normal form (i.e., *Put the ball in the box!*), and after a random delay of 200-500 ms, the hand icon automatically started moving towards the target and navigated the target object to the box. In a pilot study, for each target location, we recorded three paths for the movement of the hand icon and the object when they were controlled by human participants (i.e., in perform trials). For each repetition, one of the three adequate paths was randomly selected. The position of the hand icon and the target object was updated at each frame according to the coordinate sequence of the selected path. Similarly to perform trials, successful action completion was signalled by the disappearance of the distractor objects (for 1500 ms), and after that, a 500-ms empty screen was presented. As for the perform condition, each trial consisted of four repetitions of the action. Participants had to click on the robot icon at the start of each repetition to initiate the execution of the action, whereas clicking on the target was only required for the first iteration.

In *read trials*, the objects appearing on the screen were the same as in perform trials, except for the hand icon (i.e., box, target, distractors, and action statement). However, none of the objects could be moved. The presentation time of objects in read trials was determined by the sum of three intervals, each based on different components of the task in the command condition: (1) A random 1200-1880 ms interval, based on the time it took to click on the robot icon in the command condition of a pilot study, (2) a random 200-500 ms interval similar to the “AI reaction time” of the command condition, and (3) the duration of a randomly chosen path from the command condition.

To ensure that participants attended action statements and target objects, they were asked to perform a mismatch detection task during the experiment. Participants received the instruction that some trials would feature a mismatch between the target object described in the statement, and the objects actually available on the screen. Eight mismatch sets were created, with a standard statement, a standard image, a mismatch statement, and a mismatch image. The eight mismatched objects/statements were the same for all participants. Mismatch trials were randomly intermixed with experimental trials. Similar to experimental trials, mismatch sets were also presented twice. In one of the runs, only the standard statement and the standard image were presented; thus, the mismatch trial was identical to the experimental trials. In the other run, however, one of the four repetitions was a mismatch repetition, where either the standard statement or the standard image was replaced with a mismatch object. In such instances, participants had to signal the mismatch by pressing the space bar on the keyboard. Mismatches could appear in all three action presentation conditions and any of the four repetitions. Participants had to respond to the mismatches before the distractor objects disappeared from the screen, that is, either before or during the object collection phase in the perform and command trials, and during the corresponding time interval in the read trials. Correctly identifying a mismatch triggered a visual signal acknowledging the correct response. An error message was displayed if participants pressed the space bar when no mismatch object was presented.

##### Test phase

The second experimental session featured a recognition test that proceeded in two rounds. The first round used all 24 action statements of the first session as well as the 8 new action statements that had been set aside in the first session. Participants were shown one statement at a time, and they had to decide whether they had executed the actions themselves in the action presentation phase. The 8 standard statements/images of the mismatch trials were also displayed, but these were not included in statistical analyses. In each trial, the screen showed the action statement (font size of 0.05 height units) and a picture of the target object below (size of 0.13 × 0.13 height units). The question “Did you perform the action?” appeared above the statement. Two response options were displayed in the lower half of the screen “Yes, I performed it” (on the left), and “No, I did not perform it” (on the right). Participants were instructed to select the first option if they had executed the action themselves, that is, if the action was presented as a “perform” trial in the first phase. Participants responded by left-clicking the relevant option on the response screen. Selections could be finalised by clicking the “Continue” button. Before that was done, participants could change the selected response. The 40 statements (including mismatch objects) were presented in random order, separated by a blank screen of 500 ms.

The second round of the test phase repeated all items that had been categorised as “not performed” in the first round. The round was similar to the first one, with two important differences: The question at the top of the screen was changed to: “How did you encounter the following action?” At the bottom of the screen three options were presented (in the same order from left to right): “commanded,” “read,” and “new.” Participants were instructed to select “commanded” if they had commanded SAM to execute the action, “read” if they had read the statement without performing the action, and “new” if the action statement and the object had not been presented during the first session. The order of the items presented in the second round was randomised. The second round of the source attribution task was only announced after finishing the first round to make the first round as similar as possible to the test phase of the previous studies.

#### Data acquisition

The experiment was written in *PsycoJS* and uploaded to the *Pavlovia* website. Participants completed the experiment online, using their own computer. They were asked to use a mouse (not touchpad) and to open the experiment in full-screen mode. The action presentation and the test phase were completed in two separate sessions one day apart. The first experimental session was preceded by an instruction and practice phase. Participants could practice how they could pick up and navigate the hand icon and the target object in the perform trials, how they could select target objects and instruct SAM to complete the action in the command trials, and how they could signal if they detected a mismatch. They also observed an example of a read trial. In these practice trials, geometric shapes were used instead of the object images that were presented later in the experimental trials. Before each round of the second experimental session, participants received instructions on what the response options meant and how they could mark the selected response. In both sessions, the experimental phase started immediately after practice/instruction. Participants completed each session without pause. The first session took ca. 35 minutes, the second session ca. 5 minutes to complete. Participants were not informed beforehand what type of task they could expect in the second session, the recognition test was only revealed at the start of the session.

#### Statistical analysis

In the action presentation phase, we calculated the average exposure duration (length of a repetition) for each participant and condition. This duration was determined as the time between the action statement and the target object appearing on the screen and their disappearance. This included the time required for initiating the action (by picking up the hand icon or by clicking on the robot icon), the time required for collecting the target object, and the 1.5 seconds where the distractors disappeared and only the action statement and the target object were displayed on the screen. Each presentation trial consisted of four iterations and each item was presented twice during the first session. The total time during which participants saw the target and the action, therefore, was the sum of eight individual exposure durations. We compared exposure durations between the three conditions using a repeated-measures analysis of variance (ANOVA). For pairwise comparisons, we used two-tailed *t*-tests for paired samples with Holm correction (denoted as *p_H_*). We also assessed participants’ performance on the mismatch detection task by calculating the number of mismatch items that they recognised and the number of cases where a mismatch was falsely reported. We report the group mean and standard deviation for these values.

The interpretation of participants’ responses in the second test round is ambiguous, since these are conditional on the first-round responses. Thus, in the main text, we only report analyses related to the first test round. We calculated the percentage of “performed” responses for each participant and for each of the three action presentation conditions of the first phase and for new items as well. (i.e., What percentage of the 8 items in a condition was recognised as performed?) These values were submitted to a repeated-measures ANOVA. Pairwise comparisons were conducted with two-tailed *t*-tests for paired samples. We did not apply a correction for multiple testing for our main test, i.e., for the planned comparison of *commanded recognised as performed* and *read recognised as performed* errors. For the remaining pairwise comparisons, we report *p*-values corrected by the Holm method (*p*_H_). We expected that even with a single presentation phase, *commanded recognised as performed* errors should be more frequent than *read recognised as performed* errors. Observing this pattern of false alarms would confirm that observation inflation is indeed due to a specific link between performance and observation and not by confounds that stem from the complex structure of the three-phase paradigm.

Analyses were performed in *R* (version: 3.6.3, R Core Team, 2020). We used the *pwr* (version: 1.3-0, Champely, 2020) package for the power analysis. Processing and cleaning the raw data drew on the packages *tidyverse* (version: 1.3.0, Wickham, 2019), *Rmisc* (version: 1.5.1, Hope, 2022), and *schoRsch* (version: 1.9.1, Pfister & Janczyk, 2020), whereas statistical analyses were implemented with the packages *ez* (version: 4.4-0, Lawrence, 2016), *BayesFactor* (version: 0.9.12-4.2, Morey & Rouder, 2018), and *bayestestR* (version: 0.11.5, Makowski et al., 2019). The *ggplot2* package (version: 3.3.5, Wickham, 2016) was used for visualising the results.

### Results

#### Action presentation phase

The ANOVA showed a significant difference between exposure duration in the three action presentation conditions, *F*(2, 96) = 29.40, *p* < .001, η_p_^2^ = .38, *BF_10_* > 10^8^. Post hoc *t*-test indicated that exposure duration was shorter in the perform condition (*M* = 4.40 s, *SD* = 0.72 s) than in the other two conditions (both *p_H_*s < .001). Exposure duration in the command condition (*M* = 5.28 s, *SD* = 0.88 s) was longer than in the read condition (*M* = 5.01 s, *SD* = 0.03 s) (*p_H_* = .036). During the action presentation phase, participants recognised most of the 8 mismatch trials (*M* = 7.18, *SD* = 1.20), while committing relatively few false alarms (*M* = 0.84, *SD* = 1.45).

#### Test phase

**Figure 2.**
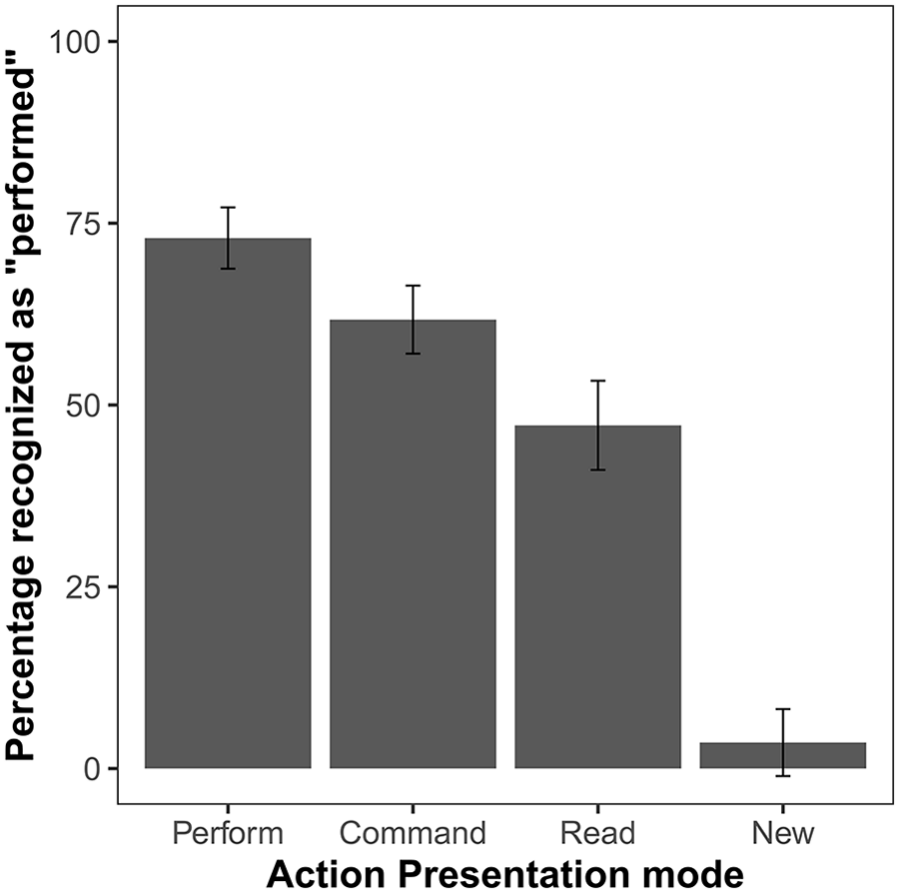
Mean percentage of *“performed”* responses in the first test round of Experiment 1. Action presentation mode distinguishes the relevant conditions during the action presentation phase (perform, command, read) as well as newly presented items. Error bars show 95% confidence intervals adjusted for within-subject designs, using the method suggested by Morey (2008).

The ANOVA comparing the percentage of items recognised as performed in each of the conditions showed a significant effect, *F*(3, 144) = 121.67, *p* < .001, η_p_^2^ = .72, *BF*_10_ > 10^38^. The planned comparison of “performed” responses for commanded and read items showed that commanded items were recognised as performed significantly more often than read ones, *t*(48) = 3.53, *p* < .001, *d* = 0.50, *BF*_10_ = 31.05. Post hoc comparisons revealed that both commanded and read actions were recognised erroneously as performed more often than new items (*p_H_*s < .003) (Figure 2, Table 1).

**Table 1.** Group average response percentages and selection times for each action presentation condition and response type in Experiment 1.

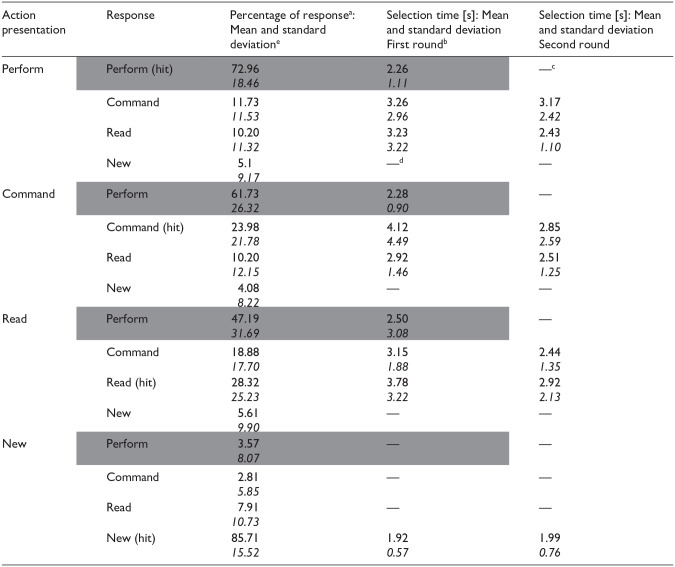

a Only “performed” responses lead to attribution to a specific source in the first round. (Categorisations solely based on the first round are presented with grey background.) In this round, all other items were categorised as “not performed.” These items were attributed to specific sources only later, in the second round of the recognition test.

b Response times are calculated separately for each hit and error type as categorised by the end of the two test rounds. However, in the first round no distinction was made between these events: They were all categorised as “not performed.” For responses other than “performed,” the first-round selection time shows the response time for this “not performed” response.

c Items categorised as “performed” were not presented in the second round.

d Because of the low number of errors involving the new items/responses, selection time was not estimated for those error types.

e Standard deviations are displayed in italics.

### Discussion

The first test round replicated the observation inflation effect even though performance and observation were included in the same phase of the experiment: False attribution to self-performance was more common for observed actions (command condition) than for action statements that were read by the participants (read condition). Although the pattern of the results was similar to those observed in previous studies, the percentage of self-attribution errors (both for observed and read items) was substantially higher than the numbers reported previously (62% vs. 12-44% for observed actions and 47% vs. 1-9% for reading). Since the number of performed responses was low for new items, we can exclude the possibility that participants simply selected the confirmatory response (“Yes, I performed it” vs “No, I did not perform it”) irrespective of the actual memory. It is possible, however, that in most instances where participants remembered that they encountered the item, but they were not confident in their recollection of the source, the formulation of the question induced a self-attribution bias, and the item was categorised as performed. While participants could reliably distinguish new and previously presented items, identifying the source of the presented items might have been more difficult in the online study than in previous experiments where actions were performed with real objects. A reason for this could be that both with regard to actions and to objects, the number of distinctive features that could support discrimination of the items was lower in the online setting. Furthermore, the sensory similarity of the three presentation conditions was also increased in the current version by all conditions being presented in the same way (on the computer screen) and from the same perspective. Combined with the possibility of a response bias, the increased difficulty of source attribution could explain the overall higher rate of “performed” responses. Another possible cause for the increased overall self-attribution tendency in comparison to previous observation inflation studies might be the difference between populations that the samples were selected from. Motivation to perform well might have been higher in the university-student samples used in previous studies than in the more diverse sample recruited from the participant pool of the Prolific website. The lower motivational level might have resulted in participants being influenced to a larger degree by the bias inherent in the binary source memory test.

Despite such differences in memory performance, the results indicate that the effect of action observation on self-attribution was similar in the online version as in previous face-to-face settings. This shows that the observation inflation effect is not caused by a bias stemming from the specific structure of the three-phase paradigm. However, without a more detailed assessment of participants’ memories, it is impossible to decide whether this effect is caused by an egocentric action appropriation tendency or by a bidirectional conflation of action-related sources (i.e., action performance and action observation). Experiment 2 set out to disentangle these two possibilities.

## Experiment 2

Experiment 1 showed that the core findings of previous observation inflation studies can be replicated with the online paradigm using only a single phase to implement the three presentation conditions (perform, observe, read). Our goal in Experiment 2 was to adjust the test phase to feature a full source memory test. The additional data could help clarify whether observation inflation is indeed an egocentric effect or if it only appears so because previous studies had selectively focused on a small number of error types. Thus, the two test rounds of Experiment 1 were compiled into a single source monitoring test that presented participants with four source options. The assessment of a wider range of source monitoring errors could contribute to a better understanding of the mechanisms of the observation inflation effect. An egocentric action-appropriation tendency would imply that observation inflation (observed recognised as self-performed errors) should be more frequent than the reverse error pattern (self-performed recognised as observed). Moreover, the difference between action observation (i.e., command condition) and reading that is characteristic of the observation inflation effect should not persist with the complementary type of source monitoring errors. That is, self-performance should be misattributed with similar frequency to action observation and reading. On the other hand, if the difference in the influence of action observation and reading on self-attribution is caused by a bidirectional conflation of observation and self-performance, error patterns should be symmetrical: The observation inflation effect should be complemented by a self-performance inflation effect. That is, self-performed actions should be attributed more often to action observation than to reading. Furthermore, the conflation of self-performed and observed actions should be reflected in the similar frequency of observed recognised as self-performed and self-performed recognised as observed errors.

### Method

#### Participants

Participants were recruited and instructed as in Experiment 1. The sample size was based on the same power analysis as Experiment 1, but in this case, we did not set the number of participants invited to the first session, instead, we decided to collect data until the sample size exceeded 56 (after drop-out and application of rejection criteria). This target sample size and further methodological decisions were preregistered at https://aspredicted.org/6JT_197. Ninety-one young adult volunteers signed up for the study. Seventeen participants only completed the first session: Nine of them decided not to participate in the second session and eight were not invited because they failed the attention check of the first session. Of the 74 participants who completed the second session, 9 were not included in the statistical analyses, because they did not meet predetermined inclusion criteria. The final sample consisted of 65 participants (mean age: 26.9 years, range: 18-35 years, 18 men, 47 women).

#### Stimuli, procedure

Stimuli and procedure were the same as in Experiment 1, with the only difference being that the test phase now featured a full source monitoring test (administered in a single round). In this test phase, object and action statement appeared on screen as in Experiment 1, the only modification was that all four possible action sources were presented as response options. The options “performed,” “commanded,” “read,” and “new” (always in this order) were presented below the object image in each trial.

#### Statistical analysis

First, we conducted the same repeated-measures ANOVA comparing the percentage of “performed” responses for the four item types as in Experiment 1. We further explored differences between conditions with paired *t*-tests. Then we performed a similar analysis (ANOVA and paired *t*-tests) on the percentage values of misattributing self-performance to one of the other three conditions. For the planned analyses, (*commanded recognised as performed* compared to *read recognised as performed* and *performed recognised as commanded* compared to *performed recognised as read*), *p*-values are reported without corrections, whereas we applied Holm corrections for all remaining comparisons.^4^

The most important analysis in Experiment 2 is the comparison of *commanded recognised as performed* and *performed recognised as commanded* errors. This analysis tests whether the high proportion of *commanded recognised as performed* errors that was observed in previous studies, and also in Experiment 1, is based on the appropriation of others’ actions, or whether it instead results from a bidirectional conflation of sources, without action memories gravitating specifically towards self-performance. To further explore this question and to confirm the pattern that emerged from the pairwise comparisons, we performed a 2 x 2 repeated-measures ANOVA with the within-subject factors conflated conditions (command/perform and read/perform) and direction of the conflation (other condition misattributed to own performance, performed action misattributed to other source). As an exploratory analysis, we assessed the correlation between the number of *commanded recognised as performed* and *performed recognised as commanded* errors.

We also compared the hit rates for items of the four conditions (ANOVA and post hoc *t*-tests with Holm-correction). Moreover, we compared the percentage of correct responses to chance level. The chance level for new items was set at 25%. However, since participants were very effective in distinguishing actually presented and new items, for perform, command, and read items, we tested against a level of 33% to credit the fact that “new” responses were almost never given as false alarms.

As in the case of Experiment 1, further analyses are reported in the Supplementary Material, i.e., an analysis of error types that are not relevant with regard to the main hypotheses and exploratory analysis using multinomial processing tree (MPT) modelling.

### Results

#### Action presentation phase

As in Experiment 1, the repeated-measures ANOVA showed a significant difference between exposure durations in the three action presentation conditions, *F*(2, 128) = 11.08, *p* < .001, η_p_^2^ = .15, *BF_10_* = 657.58. Exposure was shorter in the perform condition (*M* = 4.64, *SD* = 1.33) than in the other two conditions (perform vs. command *p_H_* < .001, perform vs. read: *p_H_* = .035). The exposure duration in the command condition (*M* = 5.33, *SD* = 1.07) was longer than in the read condition (*M* = 5.01, *SD* = 0.03) (*p_H_* = .035). During the action presentation phase, participants recognised the majority of the 8 mismatch trials (*M* = 6.88, *SD* = 1.10), while committing relatively few false alarms (*M* = 0.72, *SD* = 1.04).

#### Test phase

**Figure 3.**
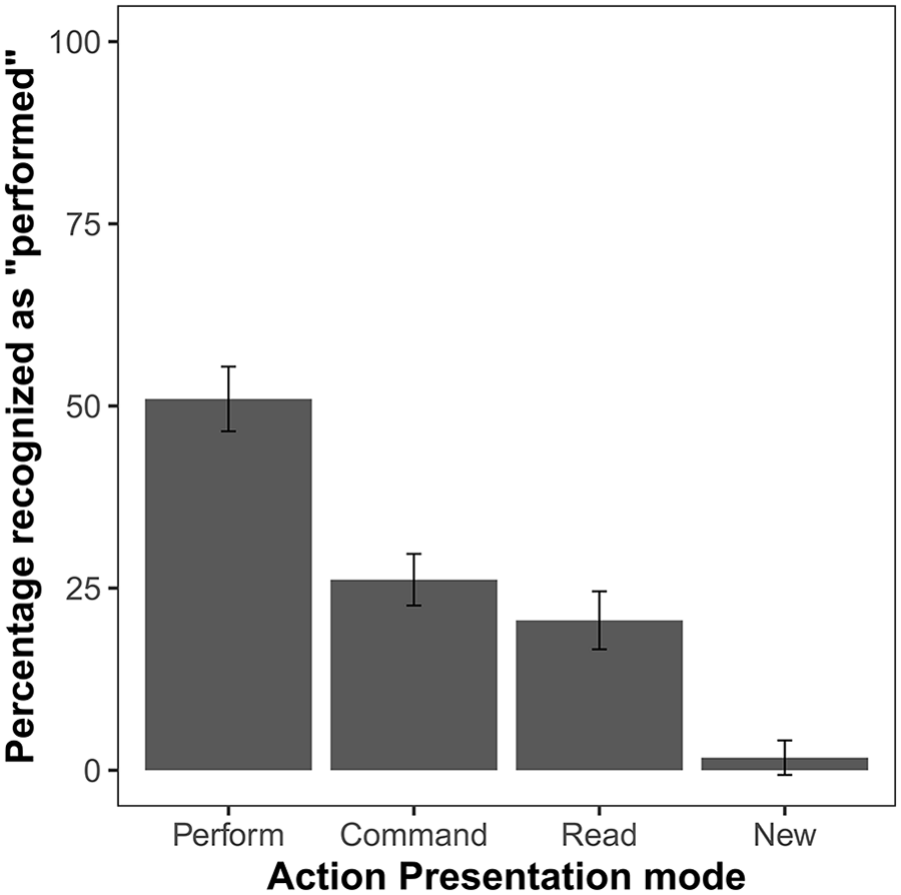
Mean percentage of *“performed”* responses in the test phase of Experiment 2. Error bars show 95% confidence intervals adjusted for within-subject designs, using the method suggested by Morey (2008).

The comparison of the percentage of “performed” responses for items presented in the four action presentation conditions showed a significant effect, *F*(3, 192) = 98.26, *p* < .001, η_p_^2^ = .61, *BF_10_* > 10^37^ (Figure 3). The planned comparison of *commanded recognised as performed* and *read recognised as performed* errors showed numerically more “performed” errors for commanded items, but this difference was not significant, *t*(64) = 1.98, *p* = .052, *d* = 0.25, *BF*_10_ = 0.81. For the remaining (post hoc) pairwise comparisons, Holm correction was used. All comparisons showed a significant effect. That is, the percentage of “performed” responses was higher for performed items than for any other action presentation condition; for new items, it was lower than for any other condition (all *p_H_*s < .001)

**Figure 4.**
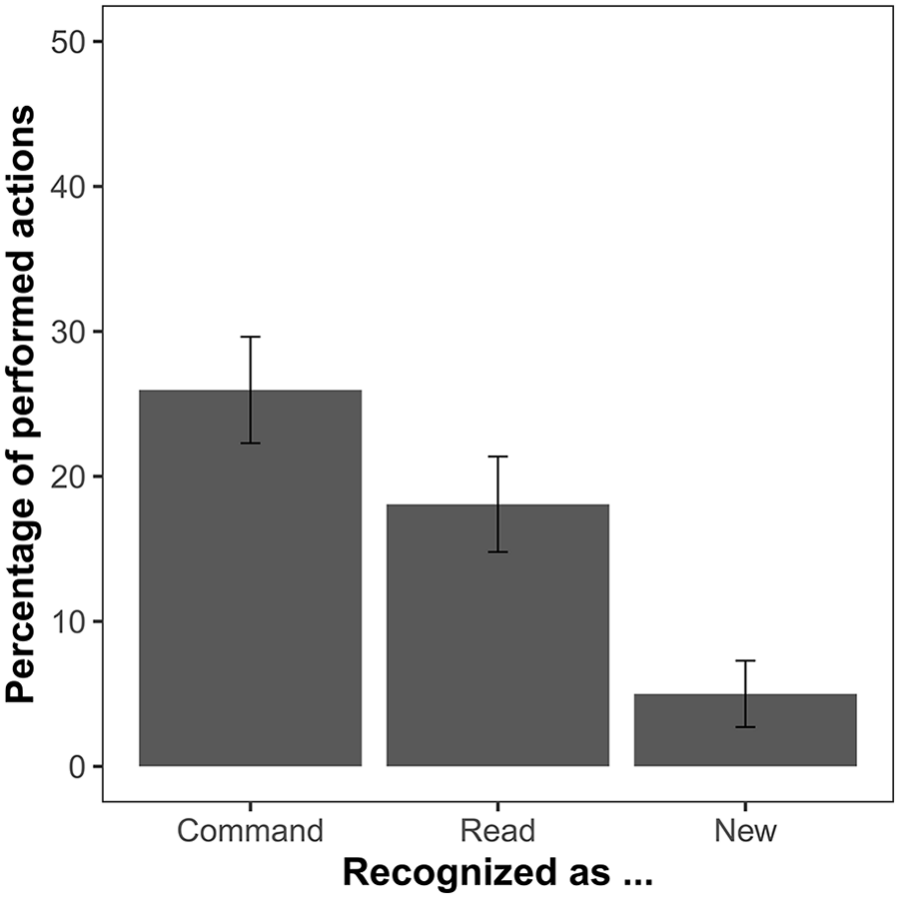
Mean percentage of action statements that were performed by the participant during the action presentation phase but not recognised as such in the test phase of Experiment 2. Error bars show 95% confidence intervals adjusted for within-subject designs, using the method suggested by Morey (2008).

The ANOVA comparing the percentage of performed actions that were recognised as commanded, read, and new also showed a significant effect, *F*(2, 128) = 32.31, *p* < .001, η_p_^2^ = .34, *BF_10_* > 10^11^ (Figure 4). The planned comparison of *performed recognised as commanded* and *performed recognised as read* errors indicated that performed actions are more often remembered as commanded than as read, *t(64)* = 2.47, *p* = .016, *d* = 0.31, *BF*_10_ = 2.25. The two additional post hoc comparisons showed that *performed recognised as commanded* and *performed recognised as read* errors are both significantly more frequent than *performed recognised as new* errors (both *p_H_*s < .001).

**Figure 5.**
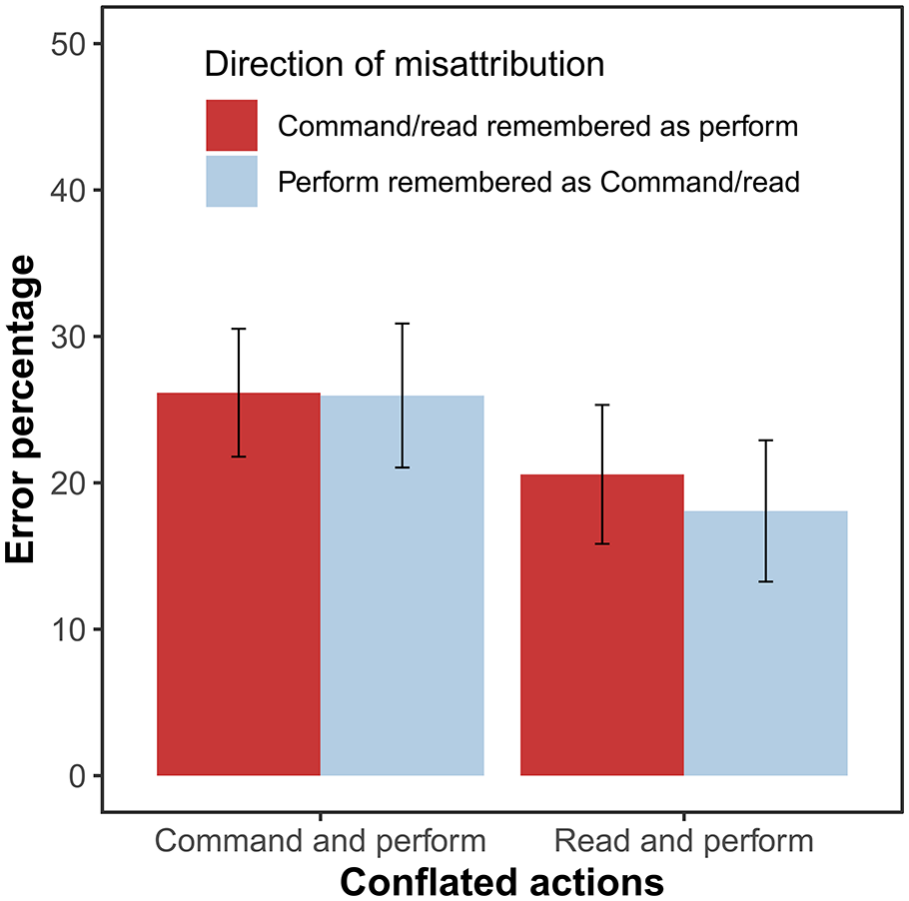
Mean percentage of errors that involve the conflation of performed and read or performed and commanded actions in Experiment 2. Error bars show 95% confidence intervals adjusted for within-subject designs, using the method suggested by Morey (2008).

We also compared whether the conflation of performed and commanded actions is characterised by the specific tendency to appropriate the other’s actions, but the error types of *commanded recognised as performed* and *performed recognised as commanded* were equally frequent, *t*(64) = 0.06, *p* = .955, *d* = 0.01, *BF*_10_ = 0.01. The 2 (conflated conditions) × 2 (direction of the conflation) ANOVA of error percentages showed a main effect of conflated conditions, *F*(1, 64) = 9.87, *p* = .003, η_p_^2^ = .13, *BF_inc_* = 8.57, indicating that the conflation of the perform and command conditions is more frequent than the conflation of perform and read conditions (Figure 5). We did not observe a main effect for direction of the conflation, *F*(1, 64) = 0.24, *p* = .629, η_p_^2^ < .01, *BF_inc_* = 0.16, nor an interaction between the two factors, *F*(1, 64) = 0.30, *p* = .588, η_p_^2^ < .01, *BF_inc_* = 0.21. The correlation between *commanded recognised as performed* and *performed recognised as commanded* errors was not significant, *r*(65) = -.04, *p* = .729, *BF*_10_ = 0.30.

The ANOVA comparing the hit rates for the four action presentation conditions showed a significant effect, *F*(3, 192) = 80.71, *p* < .001, η_p_^2^ = .56, *BF*_10_ > 10^33^. Post hoc *t*-tests tests with Holm correction indicated that the hit rate was substantially higher for new items than for the other three action presentation conditions (all *p_H_*s < .001). No difference in hit rates was observed between the other three action presentation conditions (all *p_H_*s > .98). Importantly, the hit rate was well above the chance level for all four action presentation conditions (Table 2).

**Table 2.** Group average response percentages and selection times for each action presentation condition and response type.

Action presentation	Response	Percentage of response type: Mean and standard deviation^c^	Comparison of response percentage to chance level^a^ (*t*-values)	Selection time (s): Mean and standard deviation
Perform	Perform (hit)	50.96 *21.57*	18.92***	3.05 *1.52*
	Command	25.96 *17.72*	—	3.53 *3.28*
	Read	18.08 *15.15*	—	3.60 *2.75*
	New	5 *7.91*	—	—^b^
Command	Perform	26.15 *20.34*	—	3.42 *2.39*
	Command (hit)	47.5 *20.99*	18.12***	3.62 *1.85*
	Read	23.85 *16.64*	—	4.17 *4.65*
	New	2.5 *6.33*	—	—
Read	Perform	20.58 *22.58*	—	3.54 *4.27*
	Command	26.54 *17.47*	—	3.81 *2.83*
	Read (hit)	49.23 *23.89*	16.50***	3.52 *1.78*
	New	3.65 *7.24*	—	—
New	Perform	1.73 *6.95*	—	—
	Command	2.31 *5.80*	—	—
	Read	3.65 *7.24*	—	—
	New (hit)	90.58 *13.26*	54.90***	2.32 *1.08*

*** *p* < .001.

a Chance level for correct responses in the new condition is at 25 percent, whereas we used 33.3 percent in the other three conditions.

b Because of the low number of errors involving the new items/responses selection time was not estimated for those error types.

c Standard deviations are displayed in italics.

### Discussion

With the adjustment of the source monitoring test, there was an overall reduction of the number of items that were categorised as self-performed compared to Experiment 1 (for a direct comparison of the two experiments, see the Supplementary Material). This resulted in less self-attribution errors both for observed (command condition) and read items. The results indicated a difference between *commanded recognised as performed* and *read recognised as performed errors*, but this effect was weaker than in Experiment 1 and it remained slightly below significance level. The overall decrease of performed responses was also reflected in the lower number of correctly recognised self-performed actions. There was also an increase in the number of misattribution errors for performed items, which was not equally distributed between the other two presentation conditions. Similarly to erroneous “performed” responses, a difference between observation and reading emerged: Performed actions were categorised as observed actions significantly more often than they were attributed to reading. Importantly, the higher number of *performed recognised as commanded* errors also meant that participants committed this error type with similar frequency as *commanded recognised as performed* errors. In summary, the pattern observed in Experiment 2 fits the bidirectional source conflation hypothesis perfectly: Performance is more prone to being conflated with action observation than with reading, but the conflation pattern is markedly symmetrical.

## General discussion

The two present experiments assessed potential confounding factors in research on observation inflation (Lindner et al., 2010, 2016; Schain et al., 2012; Wang et al., 2022). On the one hand, our findings confirmed that observed actions are more likely to be categorised as self-performed than new and read items. By simplifying the structure of the experiment relative to the three-phase paradigm, we could control certain factors (item memory, inadequate response options) that might have impacted previous results. Replicating the findings of previous studies with this new experimental design seems to suggest that there is indeed a tendency to remember observed actions as self-performed. On the other hand, our findings indicate that the observation inflation effect is not egocentric in nature: We did not observe a significant difference between the number of observed items recognised as self-performed and the number of self-performed items recognised as observed ones. Bayesian analysis also provided strong support for the notion that the frequency of these two error types is similar (Bayes factor of 0.01). Corresponding findings were previously interpreted as evidence against the motor simulation account (Lange et al., 2017). While we concur that the symmetrical error pattern fits better with source monitoring accounts of observation inflation (e.g., Foley et al., 2002; Foley & Ratner, 1998; Ratner et al., 2002; Sommerville & Hammond, 2007), in the following we will argue that the two views are compatible.

### Motor simulation and source monitoring

The results that remembering observation as performance and performance as observation occur with similar frequency—at least for adult participants—conform with previous findings (Lange et al., 2017). Some studies report that performance being misattributed to observation is even more frequent than the observation inflation effect (Conway & Dewhurst, 1995; Manzi & Nigro, 2008). Lange et al. (2017) suggested that the egocentric aspect of the performance-observation conflation is of essential importance to the motor simulation view of observation inflation: In the case of observed actions, motor traces serve as cues of self-performance while visual cues (second-person perspective) indicate an external source. This conflict leads to the misattribution of the observed actions. In the case of performed actions, there is no conflict, since both motor traces and visual cues (first-person perspective) suggest an internal source (performance). Based on this idea, Lange and colleagues interpreted the lack of evidence for an egocentric observation inflation effect as support for the source monitoring account over a motor-simulation-based explanation.

We do not agree with this interpretation of the results. As we see it, the motor simulation account does not stand in contrast with the source monitoring framework. It simply suggests a specific motor cue that contributes to source attribution when memories are recalled. In this regard, it is not very different from other source monitoring approaches that presume a similar source attribution mechanism but emphasise the role of other cues (e.g., perceptual similarity: Johnson et al., 1988; Thomas et al., 2003, or anticipatory processes: Foley et al., 2002; Sommerville & Hammond, 2007).

According to our findings, the issue with previous observation inflation studies is not that they have identified the wrong mechanisms of source attribution. The problem is rather that studies try to explain an egocentric effect that might not exist in the first place. As a consequence, they incorporated questionable presumptions in the interpretation of their findings: It is not elaborated why motor traces would be considered a cue of self-performance (which is a central aspect of the motor simulation account: Lindner et al., 2010, 2016). If the same motor processes are involved in action performance as well as in processing observed actions, people should have extensive experience with motor activation accompanying both event types. Thus, it is not clear why an egocentric bias in the interpretation of motor activation should occur. If we assume, however, that motor traces serve as cues that distinguish action events from non-action events, then they could explain why a bidirectional source conflation between observed and performed actions occurs while both of these event types can be distinguished more clearly from non-action events (e.g., reading). This critique also applies to other explanations of observation inflation: Anticipatory processes or perceptual richness are also not viable as cues that distinguish action performance from action observation when in many cases, these processes/features characterise both event types.

Previous results provide substantial evidence that motor processes play a central role in the observation inflation effect (Foley et al., 2002; Lindner et al., 2010, 2016; Wang et al., 2022). The current results do not question this assumption, and they are also not able to distinguish between explanations that fit this criterion (i.e., the motor simulation and the shared cognitive operation account). Instead, they indicate that studies should strive to explain bidirectional source conflation and not an egocentric observation inflation effect. (Provided that our results generalise to offline settings. This issue is discussed later.)

The assumption of bidirectional source conflation suggests that observed recognised as performed errors and performed recognised as observed errors are both source attribution errors caused by non-distinctive cues (Johnson et al., 1988). However, simply showing that these errors are committed with similar frequency does not provide conclusive evidence for a common cause. It is possible that the similar frequency of the observation inflation and self-performance inflation errors in the current study is just a coincidence, and the two error types are caused by different processes. This interpretation might be supported by the lack of correlation between the two error types. However, since the current experimental design was not planned with correlational analyses in mind and the sample size of Experiment 2 is not adequate for reliable correlational analysis, the relationship between observation inflation and self-performance inflation should be explored in the future with an experimental method that targets this question specifically.

### Item and source memory for observed actions

The complex structure of prior three-phase experiments and the limited range of source monitoring errors that were assessed in these studies left open the question of whether observation inflation is indeed an effect that exclusively reflects the unique relationship between action performance and observation, or if it also relies on contribution from less specific memory processes. Enhancement of item memory by the separate action-observation phase might result in similar data patterns to those reported in observation inflation studies. In our study, however, the performance-observation conflation effect could not be explained with such general memory effects: (1) The single presentation structure reduced the possibility of such effects. (2) Better item memory for observed than for read items was not evident in the current study: Although the number of correct “old” responses was numerically higher for observed items, the difference in comparison to the read condition was not significant. (3) The results of the main comparisons did not change if we adjusted the percentage of source monitoring errors to the number of correctly recognised old items in the corresponding condition.

Changes that we made to the three-phase paradigm allowed us to control several possible confounding factors, but others still might impacted the results in the current experimental setting. For instance, differences between observation-performance and reading-performance conflation might be explained by better overall source memory for read items. Pairwise comparison of specific source monitoring errors did not support this idea: *Commanded recognised as read* errors were not more frequent than *read recognised as commanded* errors (see Table 2 and Supplementary Material, Table S2), in fact numerically the latter error type was committed more often.

### Limitations

Since the experiment was conducted online, besides theoretically motivated modifications, the experimental paradigm also differed in some other important aspects from the original, offline version of the task. Some of these factors might also influence the generalizability of our results.

First, in contrast to previous observation inflation experiments where observed actions were executed by real human agents (either in face-to-face interactions or on video recordings), in the current design, actions of an artificial agent were used. Factors influenced by this choice (e.g., higher perceptual similarity of the conditions, and knowledge of the artificial nature of the co-actor) may have impacted the conflation of action performance and observation. However, while the effect of observing a non-human agent on source monitoring errors has not been examined in previous studies, there is substantial evidence indicating that features of human interaction, like visual stimuli that look like a human face, or movement trajectories resembling biological movement can evoke similar dispositions and behavioural patterns as actually interacting with a human partner (Elsner et al., 2012; Graf et al., 2007; Saygin et al., 2004; Vogel et al., 2021).

Second, in the current study, actions in the perform and command conditions are less distinctive compared to the actions used in previous versions of the task. Operations performed on the objects are very similar in each trial (drag and drop action). In the original paradigm, on the other hand, each action is associated with a different movement. From a theoretical perspective, however, it is ambiguous whether this difference is relevant. As previous research suggests, both performing and observing simple repetitive actions results in motor activation (e.g., Pfister et al., 2013). According to the motor simulation account, this should be sufficient for integrating (self-)performance cues with the action representations. The motor simulation account only presumes that such cues are activated when memories are recalled. It does not presuppose, however, that the motor cues can reliably distinguish between individual events during the recognition phase. The same line of thought can be applied to the shared cognitive operation explanation, which is underlined by the fact that studies based on this approach use tasks that are also characterised by non-distinctive, repetitive actions (e.g., puzzle or toy building: Foley et al., 2002; Foley & Ratner, 1998; Sommerville & Hammond, 2007). In contrast with the two motor-process-based explanations, action distinctiveness might be a relevant component of the perceptual similarity explanation: Unique action features can serve as perceptual cues of self-performance. However, as we discussed in the Introduction, this explanation is not consistent with empirical results obtained with previous versions of the observation inflation paradigm (Lindner et al., 2010, 2016), and increased reliance on such perceptual cues in the current version of the task is unlikely, because the distinctiveness of these cues is diminished.

Third, participants perform mouse movements not only in the perform but also in the command condition. This might result in self-performance cues being associated with the memory of the event, which could explain why such events are confused with actual self-performed actions during the recognition test. There are two arguments against this interpretation of the task, however: First, it is important to note that selecting the target item was only required (and possible) in the first repetition of each trial. In the subsequent repetitions, only clicking on the robot icon was necessary to initiate movement, which did not require a movement of the mouse (see Supplementary Video). Even if an association was formed between the mouse movement and the memory of the event in the command condition, this association would have been substantially weaker than the one established in the perform condition. In contrast to the actual results, this difference would have resulted in an asymmetrical error pattern. Second, even on the first trial, the target selection differed in several key aspects from the complex action that was required for guiding the objects towards the target location. Most importantly, target selection did not involve manipulation of the object. Similar selection processes before action observation have also been part of cooperative observation inflation studies (e.g., Ratner et al., 2002; Sommerville & Hammond, 2007).

Factors that have been discussed in the previous paragraphs must be taken into consideration when comparing the current results to findings reported in previous studies. Importantly, however, tendencies that were interpreted in previous research as observation inflation were replicated in the current study. Our results, therefore, challenge the predominant interpretation of the observation inflation effect: not by reporting findings that contradict previous ones, but by providing complementary data that were not available (or not considered) previously. Replicating a pattern that is analogous to previously reported observation inflation effects indicates that the results might be interpreted at face value. As we discussed earlier, there are strong arguments supporting the idea that the online task invokes similar action performance and action observation processes as previous offline versions of the experimental paradigm. However, we cannot rule out the possibility that despite the apparent replication, the current paradigm might capture a phenomenon that is different from the previously reported observation inflation effect. Even in this is the case, our findings identify an important shortcoming in the conventionally used experimental method: With the assessment of a wider range of source attribution judgements, we could show that the pattern characteristic for observation inflation can also emerge without presuming the appropriation of observed actions. This statement holds whether the replicated pattern was induced by the same or a different mechanism as in previous studies. The current results highlight that previously reported results are compatible with several alternative explanations. As a consequence, the predominant theory of observation inflation can only be confirmed by adjusting the established experimental method and by testing more nuanced predictions than the ones examined in past studies.

### Conclusion

In the current study, we adjusted an influential experimental paradigm that was used in previous observation inflation studies to control possible confounds due to separated performance and observation stages and to compare the erroneous self-attribution of observed actions to a wider range of source monitoring errors. Our result confirmed that observed actions are conflated frequently with self-performance. However, in contrast to the predominant interpretation of the observation inflation effect, our findings suggested that observation inflation is not egocentric, because self-performed actions are remembered as observed actions with similar frequency as vice versa. The results can be interpreted within the source monitoring framework which suggests that source monitoring errors are more common when two sources are similar in some aspect. In the case of performance-observation conflation, the similarity might be based on the involvement of similar motor mechanisms in action execution and action observation.

## Supplemental Material

sj-docx-1-qjp-10.1177_17470218241306743 – Supplemental material for Observation inflation as source confusion: Symmetrical conflation of memories based on action performance and observation Supplemental material, sj-docx-1-qjp-10.1177_17470218241306743 for Observation inflation as source confusion: Symmetrical conflation of memories based on action performance and observation by Bence Neszmélyi and Roland Pfister in Quarterly Journal of Experimental Psychology

## Notes

1. The interpretation of the results of Lange et al. (2017) is complicated by the fact that in Experiment 3, they did not find either self-performance inflation or observation inflation.

2. Some prior results point to the possibility that item memory might contribute to the data patterns reported in observation inflation studies. Item memory refers to recognising that an event has been presented previously, even if the source is not identified correctly. It can be boosted by repeated exposure in general and more specifically by observation (without a similar influence on source memory). For example, in the study of Lindner and Davidson (2014), the observation-related increase in the categorization of read items as performed is paralleled by the increase of correctly recognised read items. This tendency cannot be explained by the motor simulation account, but it is consistent with explanations in terms of item memory.

3. Since the experiment was conducted online, the size of the screen on which actions were viewed and performed differed between participants. Thus, object sizes and distances cannot be reported in conventional measurement units (e.g., mm, pixels, viewing angle). Instead, we report these data relative to the height of the screen.

4. To control for the impact of item memory, the planned comparisons were also conducted with the ratio of source monitoring errors and correct item memory decisions. (For example, the number of commanded recognised as performed errors was divided by the sum of commanded items recognised as commanded, performed or read.) Importantly, these analyses revealed identical effects to those obtained without correcting for item memory thus, we only report the non-corrected results.
