# Supplementary material for: Observation inflation as source confusion: Symmetrical conflation of memories based on action performance and observation
Source: Q J Exp Psychol (Hove). 2024 Dec 27;78(11):2336–54. doi: 10.1177/17470218241306743 (PMC12531383; doi:10.1177/17470218241306743)
Supplement: sj-docx-1-qjp-10.1177_17470218241306743 – Supplemental material for Observation inflation as source confusion: Symmetrical conflation of memories based on action performance and observation [file sj-docx-1-qjp-10.1177_17470218241306743.docx]

Supplementary material for:

**Observation inflation as source confusion: Symmetrical conflation of memories based on action performance and observation**

Bence Neszmélyi^1^, Roland Pfister^1,2,3^

^1^Department of Psychology III, University of Würzburg, Würzburg, Germany

^2^Department of Psychology, Trier University, Trier, Germany

^3^Institute for Cognitive and Affective Neuroscience (ICAN), Trier University, Trier, Germany

Correspondence concerning this article should be addressed to Bence Neszmélyi, Department of Psychology III, University of Würzburg, Röntgenring 11, 97070 Würzburg, Germany. Phone: +49 (0)931 31-86819

E-mail: bence.neszmelyi@gmail.com

**1. Experiment 1**

**1.1. Error types not analyzed in the main text**

In the main text, analysis focused on items that were categorized as performed in the first test round. In Table S1, we show pairwise comparisons that examine the misattribution of actual performed items to another presentation mode in the second round of the test phase, and analyses comparing responses across the two test phases. It is important to keep in mind that analyses of data collected in the second rounds of the recognition test can only be interpreted against the assumption that participants’ responses in both rounds of the recognition test were solely based on their memories of the action presentation phase and were not biased by the manner in which action source memories were assessed. As we will discuss it later, this is likely not the case, but we still report these analyses, since their comparison to results obtained with a different method (in Experiment 2) shows how the choice of source monitoring test can influence the interpretation of the result. To make this comparison between experiments possible, we included all pairwise *t*-tests for Experiment 1 that were also performed in Experiment 2.

| *Table S1. Comparison of misattribution errors not reported in the main text (Experiment 1)* | | | | | | | | | | | | |
| --- | --- | --- | --- | --- | --- | --- | --- | --- | --- | --- | --- | --- |
|  |  | P🡪C | R🡪P | P🡪R | C🡪R | R🡪C | C🡪N | N🡪C | P🡪N | N🡪P | R🡪N | N🡪R |
| C🡪P |  | **10.84**  **1.55** |  |  | **10.16**  **1.45** |  | **14.63**  **2.09** |  |  |  |  |  |
| P🡪C |  |  |  | 0.64  0.09 |  |  |  |  | **3.21**  **0.46** |  |  |  |
| R🡪P |  |  |  | **7.27**  **1.04** |  | **4.52**  **0.65** |  |  |  |  | **8.16**  **1.17** |  |
| P🡪R |  |  |  |  |  |  |  |  | 2.48  0.35 |  |  |  |
| C🡪R |  |  |  |  |  | -2.97  -0.42 |  |  |  |  |  | 1.14  0.16 |
| R🡪C |  |  |  |  |  |  |  |  |  |  | **4.57**  **0.65** |  |
| C🡪N |  |  |  |  |  |  |  | 0.93  0.13 | -0.63  -0.09 |  | -0.81  -0.12 |  |
| N🡪C |  |  |  |  |  |  |  |  |  | -0.55  -0.08 |  | **-3.06**  **-0.44** |
| P🡪N |  |  |  |  |  |  |  |  |  | 0.85  0.12 | -0.27  -0.04 |  |
| N🡪P |  |  |  |  |  |  |  |  |  |  |  | -2.31  -0.33 |
| R🡪N |  |  |  |  |  |  |  |  |  |  |  | -1.14  -0.16 |
| N🡪R |  |  |  |  |  |  |  |  |  |  |  |  |
| * P: Perform; C: Command; R: Read; N: New  ** In the cells, *t*-values for two-tailed paired *t*-tests and *d_z_* effect sizes are displayed. Significant results (after applying Holm correction) are presented in bold. Positive values indicate that percentage of the error type displayed in the column on the left side of the table is higher than percentage of the error type in the upper row. Negative values show the opposite. | | | | | | | | | | | | |

*** Results in red: For comparisons that are reported without Holm-correction in Experiment 2, no correction was applied in Experiment 1 either.

If we take the results of the second round at face value, the findings of Experiment 1 seem to support the egocentric view of the observation inflation effect: items presented in the command condition are more frequently attributed to the perform condition than items presented in the read condition (main text). The same imbalance between the command and read conditions cannot be observed in the misattribution of items presented in the perform condition: The comparison of *performed recognized as commanded* and *performed recognized as read* errors did not indicate a significant difference. Furthermore, participants attributed commanded items to the perform condition in the first round of the recognition test substantially more often than the other way around (performed recognized as commanded) in the second round of the recognition test. However, it is important to keep in mind that the responses in the second round depend on the first-round decisions, thus, a straightforward interpretation is not possible. For example, due to the test-induced bias, all (or most) of the items where participants were uncertain with regard to the source might have been attributed to self-performance in the first test round. In this a case, a hypothetical imbalance between preformed items attributed to the command and to the read condition could not be captured in the second test round—since no (or only a few) performed items with uncertain source would remain after the first round.

In Table S1, two further error types might be relevant with regard to the observation inflation effect: As we discussed in the main text, increased item memory or reduced source memory in the command condition could explain differences in the attribution of commanded and read items to self-performance without the presumption of a specific observation-performance connection being necessary. The analysis showing that there was no substantial difference in the percentage of commanded and read items that were categorized as new contradicts the item-memory-based explanation. We also did not find evidence for the general source memory deficiency (for commanded items) explanation: The percentage of commanded recognized as read errors was not significantly different from the percentage of read recognized as commanded errors. This indicates that the increased misattribution of commanded items to the perform condition—in comparison to similar errors for read items—is specifically related to self-attribution and does not reflect a general source memory deficiency.

**1.2. Hit rates**

The percentage of correct responses was calculated for each participant and action presentation condition. We used a repeated-measures ANOVA to compare the percentage of correct responses between the four action presentation conditions. Differences between conditions were further explored with post-hoc *t*-tests (with Holm correction). The ANOVA comparing the hit rates for the four action presentation conditions showed a significant effect, *F*(3, 144) = 110.82, *p* < .001, η_p_^2^ = .70, *BF*_10_ > 10^40^. Post-hoc *t*-tests with Holm correction indicated that the hit rate was substantially higher for new items than for the other three action presentation conditions and for performed items than for commanded and read items (all *p_H_*s < .001). No difference was observed in hit rates between the command and read conditions (*p_H_* = .272) (main text, Table 1). It is also important to keep in mind that hit rates are based on first round responses for perform items, and on second round responses for all other items, which makes a direct comparison impossible.

**2. Experiment 2**

**2.1. Error types not analyzed in the main text**

Similarly to Experiment 1, the percentage of various error types were also compared in Experiment 2. We performed 19 comparisons. (Two pairwise comparisons that were reported in the Supplementary file for Experiment 1 are in the main text for Experiment 2.) Results are displayed in Table S2. Since in Experiment 2, a full source monitoring test was administered in a single round, the results are more straightforward than the corresponding analyses of Experiment 1. The results clearly suggest that errors involving new items are less frequent than errors related to the mix-up of the perform, command and read conditions. However, there seems to be no significant difference in the frequency of errors that involve new items or judgements, neither in the frequency of errors where only the three action presentation conditions of the first phase are involved. This indicates that, similarly to Experiment 1, the pairwise comparisons do not provide support for explanations in terms of item memory or overall source memory^[[1]](#footnote-1)^.

| *Table S2. Comparison of misattribution errors that are not related to the main hypotheses of the study (Experiment 2)* | | | | | | | | | | | | |
| --- | --- | --- | --- | --- | --- | --- | --- | --- | --- | --- | --- | --- |
|  |  | P🡪C | R🡪P | P🡪R | C🡪R | R🡪C | C🡪N | N🡪C | P🡪N | N🡪P | R🡪N | N🡪R |
| C🡪P |  |  |  |  | 0.6  0.07 |  | **8.58**  **1.06** |  |  |  |  |  |
| P🡪C |  |  |  |  |  |  |  |  | **8.39**  **1.04** |  |  |  |
| R🡪P |  |  |  | 0.7  0.09 |  | -1.44  -0.18 |  |  |  |  | **5.63**  **0.7** |  |
| P🡪R |  |  |  |  |  |  |  |  | **6.24**  **0.77** |  |  |  |
| C🡪R |  |  |  |  |  | -0.88  -0.11 |  |  |  |  |  | **7.48**  **0.93** |
| R🡪C |  |  |  |  |  |  |  |  |  |  | **10.16**  **1.26** |  |
| C🡪N |  |  |  |  |  |  |  | 0.18  0.02 | -2.03  -0.25 |  | -0.95  -0.12 |  |
| N🡪C |  |  |  |  |  |  |  |  |  | 0.55  0.07 |  | -2.2  -0.27 |
| P🡪N |  |  |  |  |  |  |  |  |  | 2.37  0.29 | 1.22  0,15 |  |
| N🡪P |  |  |  |  |  |  |  |  |  |  |  | -2.30  -0.28 |
| R🡪N |  |  |  |  |  |  |  |  |  |  |  | -1.07  -0.13 |
| * P: Perform; C: Command; R: Read; N: New  ** In the cells, *t*-values for two-tailed paired *t*-tests and *d_z_* effect sizes are displayed. Significant results (after applying Holm correction) are presented in bold. Positive values indicate that the percentage of the error type displayed in the column on the left side of the table is higher than the percentage of the error type in the upper row, negative values show the opposite. | | | | | | | | | | | | |

**2.2. Multinomial processing tree analysis**

As an exploratory analysis, we also investigated the results with the multinomial processing tree (MPT) method, which was developed with the goal of separating the contribution of various latent cognitive processes to categorical decisions (Riefer & Batchelder, 1988). In memory research, the method has been used for separating processes of item memory, source memory and guessing in source-monitoring paradigms (e.g., Bayen et al. 2006; Bröder & Meisner, 2007). In standard MPT models of source memory all errors are attributed to guessing (see, Bayen et al, 1996 and the adaptation of these models to three sources in Keefe et al., 2002), thus such models are not suited for capturing the systematic misattribution errors that have been presumed to cause the observation inflation effect. However, standard models of source attribution have been extended to account for source attribution processes based on multiple attributes (Meiser, 2005). These complex models can capture more specific causes of source attribution errors, beside guessing, thus, they might be a good fit for the investigation of the observation inflation effect.

Partial source memory models in particular have been developed to address source monitoring tasks where multiple sources are nested within the same category. For example, if participants have to identify the source of spoken words in a task where some of the speakers share a common trait (e.g., they belong to the same gender), it is possible that participants do not recall the exact source, but they are able to remember the category that the source belongs to (i.e., they identify the gender of the speaker). Observation inflation and self-performance inflation can be interpreted as a partial source memory errors: When participants make such errors, they do not recall the exact source, but they remember the superordinate category (i.e., that the source was associated with a motor trace). Based on this, they attribute the memory to a source that shares a common feature (motor trace) with the actual source. Thus, for the present analyses, we adapted a model of partial source memory (Dodson et al., 1998), to the observation inflation task (Figure S1). In this model, the standard source recognition model is extended by three parameters^[[2]](#footnote-2)^: The *m1_p_* and *m1_c_* parameters show the probability that the motor trace is recalled as a source cue during the recollection of an event. This trace would lead to the attribution of the item to one of the action-related sources (perform, command). The proportion of such memories being recognized as performed (vs commanded) is indicated by the self-attribution parameter (*m2*). If motor traces indeed serve as cues of self-performance, as suggested by Lindner and colleagues in the motor simulation account of observation inflation (e.g., Lindner et al., 2016), this parameter should have the value 1. (i.e., Motor traces always result in the item being categorized as performed.) In the main text, we suggested that motor traces might only signal that an event included an action, without specifying the agent. In this case motor traces could result both in performed and in commanded categorizations and in a *m2* parameter of ca. 0.5. Regarding a motor trace being associated with the action, we allowed different probabilities for performed and commanded actions (i.e., separate *m1_p_* and *m1_c_* parameters), but we assumed that the proportion of performed and commanded categorizations induced by the motor traces would be the same in the two conditions. (i.e., We did not have separate *m2* parameters for perform and command items.)

| *Table S3. Aggregated response frequencies for the four item types* | | | | | | | | | | | | | | | |
| --- | --- | --- | --- | --- | --- | --- | --- | --- | --- | --- | --- | --- | --- | --- | --- |
| Perform items | | | | Command items | | | | Read items | | | | New items | | | |
| P | C | R | N | P | C | R | N | P | C | R | N | P | C | R | N |
| 265 | 135 | 94 | 26 | 136 | 247 | 124 | 13 | 107 | 138 | 256 | 19 | 9 | 12 | 28 | 471 |
| Note:  Individual response frequencies for certain item-response combinations are very low, thus, MPT-models cannot be applied to individual data sets in the current study.  P: Recognized as perform; C: Recognized as command; R: Recognized as read; N: Recognized as new | | | | | | | | | | | | | | | |


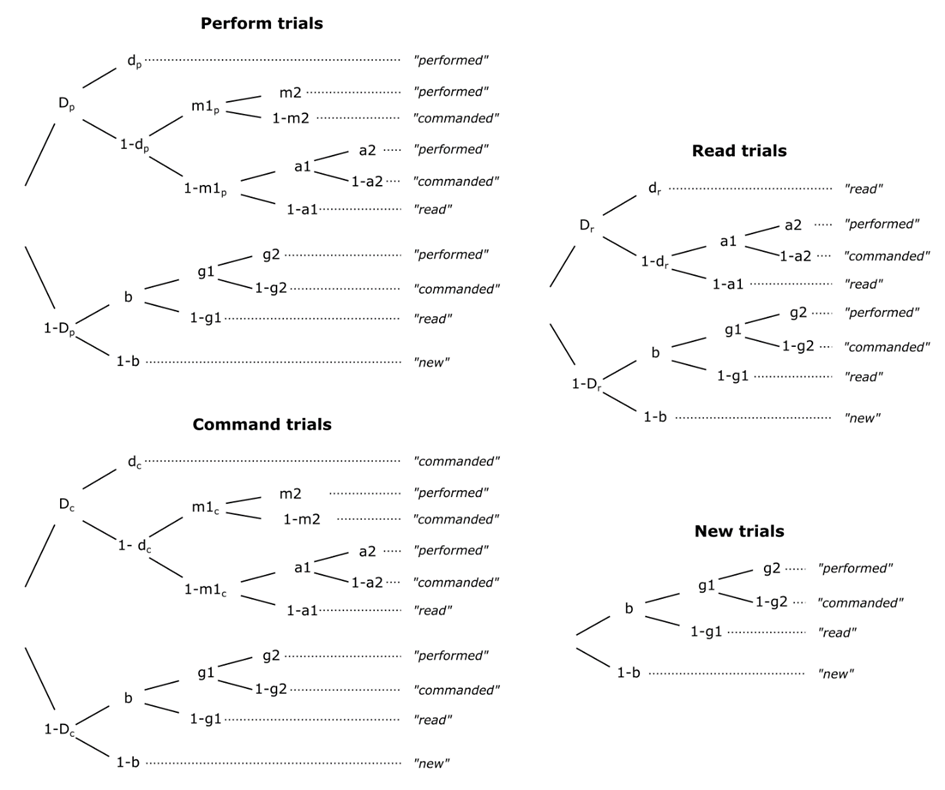


**Figure S1.** The MPT model used for the analysis of the response frequencies. The model consists of four trees for the four different item types presented in the test phase. On the right-hand side of the trees, the responses are displayed that are the results of the latent cognitive processes represented by the branching of the tree. Parameters *D_p_*, *D_c_* and *D_r_* reflect item memory (recognition) for each of the four item types and parameters *d_p_*, *d_c_* and *d_r_* correct source identification for the same item types. Selection probabilities for recognition without source identification are reflected in the *a1* (read vs perform/command) and *a2* (perform vs command) parameters while guessing probabilities for not recognized items are represented by the *G* (presented vs not presented in the learning phase), *g1* (read vs perform/command), and *g2* (perform vs command) parameters. The *m1_p_* and *m1_c_* parameters show the probability that a motor trace is stored as part of the memory, and the *m2* parameter shows the probability that this motor trace results in the categorization of the action as performed (vs commanded).

When assessing the results of the analyses, it must be taken into account that the study was not designed with MPT modelling in mind, resulting in several limitations: (1) Although, models of partial source memory have been validated (Dodson et al., 1998, Meiser, 2005) this was not done in the context of observation inflation. Thus, without re-validating the models for this specific task, it cannot be taken for granted that parameters indeed reflect the targeted processes. (2) The number of model parameters that can be investigated is limited—among others—by the number of response categories used in the source monitoring test. To be able to obtain an identifiable model, we had to put restrictions on the model parameters: Source memory was assumed to be equal for all three presentation conditions (perform, read, command). (3) Some error types occur with a very low frequency, thus, in some cases, large confidence intervals indicate a wide range of possible parameter values. Despite these limitations, we present the results of the analyses, since they can provide preliminary information on whether the MPT approach is suitable for the assessment of observation inflation, and they might serve as a starting point for future investigations. Analyses were performed with the *MPTinR* package (version: 1.14.1; Singmann & Kellen, 2013).

As a substitute for a proper validation, we examined if the inclusion of parameters targeting observation inflation and self-performance inflation indeed improves model fit. A nested model where the *m1* parameters were set to 0 was compared to the baseline model (depicted in Figure S1). The parameter restrictions resulted in a significantly worse model fit, *G^2^*(3) = 10.17, *p* = .017, highlighting the contribution of the motor trace parameters to capturing participants’ response tendencies. This analysis cannot replace validation of the model, which should be based on testing the selective influence of specific underlying processes on the model parameters—by a systematic manipulation of the associated variables. In the context of observation inflation, this could be implemented by testing the source attribution task under varying levels of motor interference (e.g., Lang et al., 2017; Lindner et al. ,2016; Wang et al., 2022).

For parameter estimates and model fit, see Table S4. The item memory parameter is close to 1 for all presentation modes, indicating that the recognition of previously presented items (regardless whether the source is identified correctly) is close to perfect. To assess if item memory had an influence on the observed response patterns, we compared a nested model with an equality restriction on the three item memory parameters (*D_p_* = *D_c_* = *D_r_*) to the baseline model. The decrease in model fit was not significant, *G^2^*(2) = 4.60, *p* = .10, indicating that the contribution of item memory to response patterns in the current observation inflation study is not substantial.

The approach of using separate motor-trace parameters for perform and command items was supported by a significant decline in model fit (compared to the baseline model) if equality restrictions (*m1_p_ = m1_c_*) were put on these parameters, *G^2^*(1) = 4.50, *p* = .034. A decline in model fit was also observed if either *m1_p_*, *G^2^*(1) = 8.93, *p* = .003, or *m1_c_*, *G^2^*(1) = 4.11, *p* = .043, was set to 0, which indicates that motor traces contribute to source attribution for performed as well as for observed actions.

Setting the self-attribution parameter (*m2*) to 0, *G^2^*(1) = 8.93, *p* = .003, or to 1, *G^2^*(1) = 4.42, *p* = .035, also resulted in a worse model fit, indicating that motor traces can be interpreted as indicators of both self-performance and action observation—and as such, they might result in performed and commanded categorizations as well. Setting the parameter to 0.5 (meaning that motor traces lead to performed and commanded categorizations with equal probability) did not result in a significantly reduced model fit compared to the baseline model, *G^2^*(1) = 3.81, *p* = .051.

| *Table S3. MPT model parameters for the baseline model (full model).* | | | |
| --- | --- | --- | --- |
| Parameter | Description | Parameter estimate | 95% CI |
| D_p_ | Item memory for performed items | .94 | [.92 - .97] |
| D_c_ | Item memory for commanded items | .97 | [.96 - .99] |
| D_r_ | Item memory for read items | .96 | [.94 - .98] |
| d_p_ | Source memory for performed items | .21 | [.16 - .26] |
| d_c_ | Source memory for commanded items | .21 | [.16 - .26] |
| d_r_ | Source memory for read items | .21 | [.16 - .26] |
| a1 | Guessing parameter 1 for items with item memory and without source memory (perform/command vs. read) | .62 | [.56 - .68] |
| a2 | Guessing parameter 2 for items with item memory and without source memory (perform vs. command) | .44 | [.37 - .50] |
| G | Guessing parameter 1 for items without item memory (“old” vs. “new”) | .10 | [.06 - .12] |
| g1 | Guessing parameter 2 for items without item memory (perform/command vs. read) | .43 | [.29 - .57] |
| g2 | Guessing parameter 3 for items without item memory (perform vs. command) | .43 | [.22 - .64] |
| m1_c_ | Motor trace recognition for performed items | .37 | [.19 - .55] |
| m1_c_ | Motor trace recognition for commanded items | .18 | [.02 - .34] |
| m2 | Source attribution for items with motor trace | .66 | [.50 - .82] |
| *df* | | 0 | |
| *G^2^* | | 0 | |
| *AIC* | | 24 | |
| ^2^Values displayed in red have equality restrictions on them. | | | |

In conventional MPT models of source monitoring, corresponding guessing parameters for items with (*a1*, *a2*) and without item memory (*g1*, *g2*) are often equated. We also compared a nested model with equality restrictions on these parameters (i.e., *a1* = *g1*, *a2* = *g2*) to the baseline model. The restrictions resulted in a significantly reduced model fit, *G^2^*(2) = 6.19, *p* = .045, suggesting that in the current data set, guessing processes are influenced by recognizing the item as one that has been presented in the first phase. The guessing parameters of the baseline model suggest a reduced tendency to categorize items as read when participants remember that the item was presented and only use guessing to determine the exact source, compared to items where participants rely completely on guessing for “old” vs “new” categorization and for source attribution as well.

In the model adopted from partial source memory studies, the motor trace parameter for the perform condition (*m1_p_*) was higher than the corresponding parameter for the command condition (*m1_c_*). This indicates that a motor trace being associated with the memory of an event is more common when actions are performed than when they are observed. The analyses also suggested that both observation inflation and self-performance inflation could contribute to the data pattern observed in our study (i.e., excluding either of these processes by setting the self-attribution parameter *m2* to 0 or 1 significantly reduced model fit). Furthermore, despite the similar number of *commanded recognized as performed* and *performed recognized as commanded* errors (see main text) the self-attribution parameter was well above 0.5, which suggest that in cases where participants make source attribution based on the motor trace, they are more likely to categorize an action as self-performed than as observed. This might point to an egocentric tendency in source attribution errors induced by motor traces. However, the difference in model fit between the baseline model (free self-attribution parameter) and the nested model with the self-attribution parameter fixed at 0.5 remained slightly above the significance threshold. Thus, for conclusive evidence, further investigation is necessary.

Beside exploring the role of motor traces in observation inflation, the MPT models also made the assessment of item-memory-related effects possible. We did not observe substantial differences in item memory that could contribute to the observation inflation effect. Somewhat surprisingly, the guessing parameters indicted a difference in response tendencies for items with and without item memory. This effect might be explained by participants’ assumption that they have better recollection of action-related events. That is, if they remember an event without recalling the source, they might assign it rather to an action-related source (self-performance, observation) than to a non-action source (reading). However, at the current stage, this explanation is speculative. As the next step, future studies could explore whether this bias also remains if source memory parameters were allowed to vary freely across presentation modes.

**3. Comparison of the two experiments**

To explore how the type of recognition test affects the tendency to misreport actions from various sources as actions performed by oneself, we compared the percentage of “performed” responses across the two experiments (Figure S2). In both experiments, the percentage of “performed” responses was calculated for each participant and action presentation condition. These values were submitted to a 2 × 4 ANOVA, with Experiment (Experiment 1, Experiment 2) as between-subjects factor and Action presentation condition (Perform, Command, Read, New) as within-subject factor. This analysis revealed a significant main effect of Experiment, *F*(1, 112) = 66.61, *p* < .001, η_p_^2^ = .37, *BF_inc_* > 10^9^, a significant main effect of Action presentation condition, *F*(3, 336) = 218.83, *p* < .001, η_p_^2^ = .66, *BF_inc_* > 10^70^, and a significant Experiment × Action presentation interaction, *F*(3, 336) = 18.04, *p* < .001, η_p_^2^ = .14, *BF_inc_* > 10^8^. To further explore the difference between the two experiments, we compared the percentage of “performed” responses in Experiment 1 and 2 in each Action presentation condition. For new items the difference was not significant (*p_H_* = .204), but in all other action presentation conditions the percentage of “performed” responses was higher in Experiment 1 than in Experiment 2 (*p_H_*s < .001). Considering only the three conditions that included items that were presented in the action presentation phase, numerically, the difference between “performed” responses in Experiment 1 and 2 was largest in the command condition, followed by the read and then the perform condition. This pattern was confirmed by assessing the Experiment × Action presentation condition interaction in post-hoc 2 × 2 ANOVAs. The comparison of the Perform and Command conditions revealed a significant effect (*p_H_* = .020), while the comparison of Perform and Read (*p_H_* = .419) and Command and Read (*p_H_* = .132) conditions did not show a significant interaction.


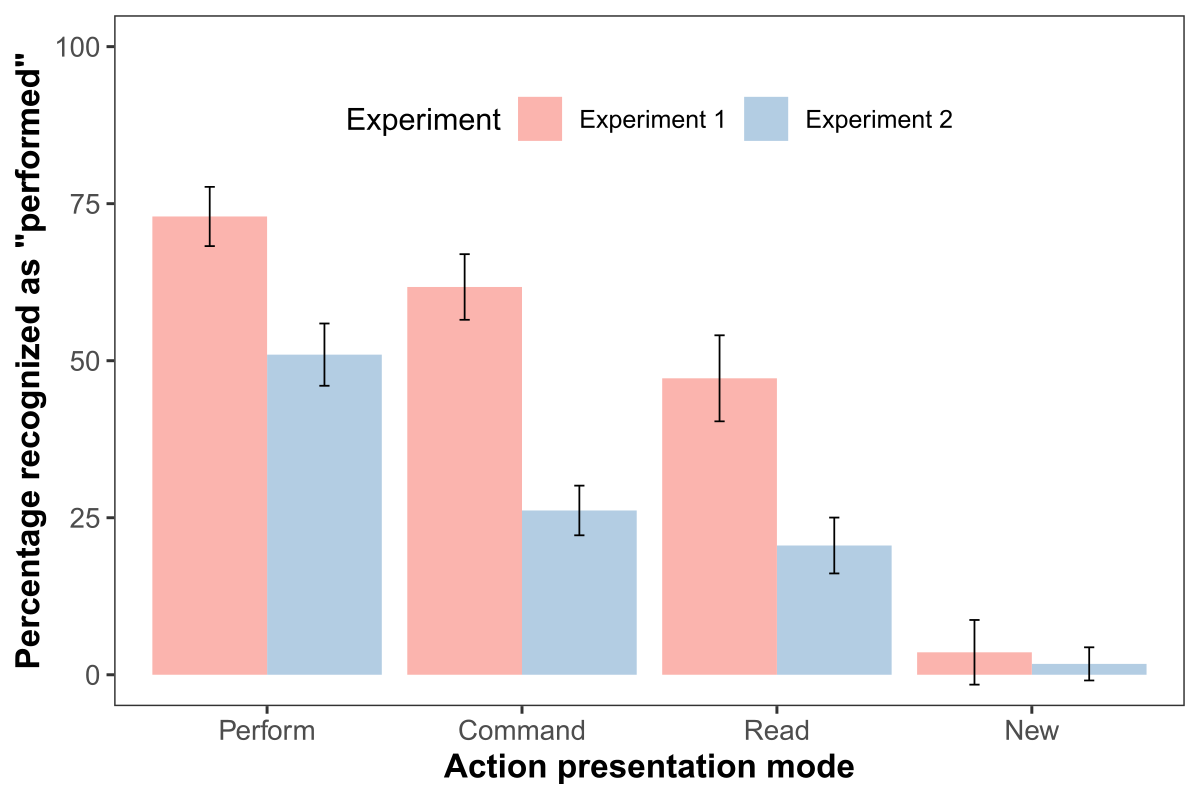


**Figure S2**. Mean percentage of “performed” responses in the test phases of Experiments 1 and 2 for items presented in various conditions during the action presentation phase. Error bars show 95% confidence intervals adjusted for within-subject factors, using the method suggested by Morey (2008).

The results clearly show that the choice of memory test can influence the results obtained in observation inflation studies. Several different test versions were used in past research—from binary decision tasks (Kashihara et al, 2017; Lindner et al., 2010; Pfister et al., 2017) and more detailed source monitoring tests (Lindner et al., 2016; Schain et al., 2012; Wang et al., 2022), over two-round recognition tasks (old-new recognition task followed by a source monitoring test: Lindner & Davidson, 2014) to a recall test in Lange et al. (2017)—which might explain some of the contradictory findings. The fact that it was the observed items (command condition) that were affected to the largest degree by the test-induced self-attribution bias is consistent with the bidirectional source conflation explanation of observation inflation, but since the effect on the command and read condition did not differ significantly, this finding cannot be regarded as strong evidence and needs further examination.

**References**

Bayen, U. J., Erdfelder, E., & Murnane, K. (1996). Source discrimination, item detection, and multinomial models of source monitoring. *Journal of Experimental Psychology: Learning, Memory, and Cognition, 22*(1), 197–215. <https://doi.org/10.1037/0278-7393.22.1.197>

Bröder, A., & Meiser, T. (2007). Measuring source memory. *Journal of Psychology*, *215*(1), 52–60. <https://doi.org/10.1027/0044-3409.215.1.52>

Dodson, C. S., Holland, P. W., & Shimamura, A. P. (1998). On the recollection of specific- and partial-source information. *Journal of Experimental Psychology: Learning, Memory, and Cognition, 24*(5), 1121-113. <https://doi.org/10.1037/0278-7393.24.5.1121>

Kashihara, S., Kanayama, N., Miyatani, M., & Nakao, T. (2017). Attentive observation is essential for the misattribution of agency to self-performance. *Frontiers in Psychology*, *8*, 890. <https://doi.org/10.3389/fpsyg.2017.00890>

Keefe, R. S. E., Arnold, M. C., Bayen, U. J., McEvoy, J. P., & Wilson, W. H. (2002). Source-monitoring deficits for self-generated stimuli in schizophrenia: Multinomial modeling of data from three sources. *Schizophrenia Research, 57*(1), 51–67. <https://doi.org/10.1016/S0920-9964(01)00306-1>

Lange, N., Hollins, T. J., & Bach, P. (2017). Testing the motor simulation account of source errors for actions in recall. *Frontiers in Psychology*, *8*, 1686. <https://doi.org/10.3389/fpsyg.2017.01686>

Lindner, I., & Davidson, P. S. R. (2014). False action memories in older adults: Relationship with executive functions? *Aging, Neuropsychology, and Cognition*, *21*(5), 560–576. <https://doi.org/10.1080/13825585.2013.839026>

Lindner, I., Echterhoff, G., Davidson, P. S. R., & Brand, M. (2010). Observation inflation: Your actions become mine. *Psychological Science*, *21*(9), 1291–1299. <https://doi.org/10.1177/0956797610379860>

Lindner, I., Schain, C., & Echterhoff, G. (2016). Other-self confusions in action memory: The role of motor processes. *Cognition*, *149*, 67–76. <https://doi.org/10.1016/j.cognition.2016.01.003>

Meiser, T. (2005). A Hierarchy of multinomial models for multidimensional source monitoring. *Methodology*, *1*(1), 2–17. <https://doi.org/10.1027/1614-1881.1.1.2>

Pfister, R., Schwarz, K. A., Wirth, R., & Lindner, I. (2017). My command, my act: Observation inflation in face-to-face interactions. *Advances in Cognitive Psychology*, *13*(2), 177–187. <https://doi.org/10.5709/acp-0217-7>

Riefer, D. M., & Batchelder, W. H. (1988). Multinomial modeling and the measurement of cognitive processes. *Psychological Review, 95*(3), 318–339. <https://doi.org/10.1037/0033-295x.95.3.318>

Schain, C., Lindner, I., Beck, F., & Echterhoff, G. (2012). Looking at the actor’s face: Identity cues and attentional focus in false memories of action performance from observation. *Journal of Experimental Social Psychology*, *48*(5), 1201–1204. <https://doi.org/10.1016/j.jesp.2012.04.003>

Singmann, H., & Kellen, D. (2013). MPTinR: Analysis of multinomial processing tree models in R. *Behavior Research Methods*, *45*(2), 560–575. <https://doi.org/10.3758/s13428-012-0259-0>

Wang, L., Chen, Y., & Yue, Y. (2022). Is motor activity the key to the observation-inflation effect? The role of action simulation. *Memory & Cognition*, *50*(5), 1048–1060. <https://doi.org/10.3758/s13421-021-01259-x>

1. On the one hand, the item-memory-based explanation would suggest that item memory is better for commanded items than for read items (lower percentage of erroneous new categorization). An explanation in terms of overall source memory, on the other hand, would entail that command actions are more often attributed to any of the other two presentation conditions than read items. That is, in addition to the difference in false “performed” attributions reported in the main text, we could also expect a higher percentage of commanded recognized as read compared to read recognized as commanded errors. [↑](#footnote-ref-1)
2. For a definition of all model parameters, see Table S3. [↑](#footnote-ref-2)
